# Implementation of child-centred outcome measures in routine paediatric healthcare practice: a systematic review

**DOI:** 10.1186/s12955-023-02143-9

**Published:** 2023-07-03

**Authors:** Hannah May Scott, Debbie Braybrook, Daney Harðardóttir, Clare Ellis-Smith, Richard Harding, On behalf of C-POS, AK Anderson, Jo Bayly, Lydia Bate, Myra Bluebond-Langner, Debbie Box, Katherine Bristowe, Rachel Burman, Lizzie Chambers, Lucy Coombes, Alan Craft, Fin Craig, Aislinn Delaney, Jonathan Downie, Julia Downing, Bobbie Farsides, Sara Fovargue, Lorna Fraser, Jane Green, Jay Halbert, Julie Hall-Carmichael, Irene Higginson, Michelle Hills, Mevhibe Hocaoglu, Vanessa Holme, Gill Hughes, Jo Laddie, Angela Logun, Eve Malam, Steve Marshall, Linda Maynard, Andrina McCormack, Catriona McKeating, Lis Meates, Fliss Murtagh, Eve Namisango, Veronica Neefjes, Cheryl Norman, Sue Picton, Christina Ramsenthaler, Anna Roach, Ellen Smith, Michelle Ward, Mark Whiting

**Affiliations:** Florence Nightingale Faculty of Nursing Midwifery and Palliative Care, Cicely Saunders Institute, King’s College London, Bessemer Rd, SE5 9RS, London, UK

**Keywords:** Implementation science, Paediatrics, Patient reported outcome measures, Systematic review, Child health services

## Abstract

**Background:**

Person-centred outcome measures (PCOMs) are commonly used in routine adult healthcare to measure and improve outcomes, but less attention has been paid to PCOMs in children’s services. The aim of this systematic review is to identify and synthesise existing evidence of the determinants, strategies, and mechanisms that influence the implementation of PCOMs into paediatric healthcare practice.

**Methods:**

The review was conducted and reported in accordance with PRISMA guidelines. Databased searched included CINAHL, Embase, Medline, and PsycInfo. Google scholar was also searched for grey literature on 25^th^ March 2022. Studies were included if the setting was a children’s healthcare service, investigating the implementation or use of an outcome measure or screening tool in healthcare practice, and reported outcomes relating to use of a measure. Data were tabulated and thematically analysed through deductive coding to the constructs of the adapted-Consolidated Framework for Implementation Research (CFIR). Results were presented as a narrative synthesis, and a logic model developed.

**Results:**

We retained 69 studies, conducted across primary (*n* = 14), secondary (*n* = 13), tertiary (*n* = 37), and community (*n* = 8) healthcare settings, including both child self-report (*n* = 46) and parent-proxy (*n* = 47) measures. The most frequently reported barriers to measure implementation included staff lack of knowledge about how the measure may improve care and outcomes; the complexity of using and implementing the measure; and a lack of resources to support implementation and its continued use including funding and staff. The most frequently reported facilitators of implementation and continued use include educating and training staff and families on: how to implement and use the measure; the advantages of using PCOMs over current practice; and the benefit their use has on patient care and outcomes. The resulting logic model presents the mechanisms through which strategies can reduce the barriers to implementation and support the use of PCOMs in practice.

**Conclusions:**

These findings can be used to support the development of context-specific implementation plans through a combination of existing strategies. This will enable the implementation of PCOMs into routine paediatric healthcare practice to empower settings to better identify and improve child-centred outcomes.

**Trial registration:**

Prospero CRD 42022330013.

**Supplementary Information:**

The online version contains supplementary material available at 10.1186/s12955-023-02143-9.

Contributions to the literature

- This systematic review provides a theory-driven appraisal of the evidence for implementing PCOMs into paediatric healthcare using the adapted-CFIR.
- Strategies to address barriers to implementing PCOMs include: 1) educating professionals, children and families on using PCOMS and the benefit they have on patient care; 2) addressing logistical and resource barriers, including time, staffing, and provision of funding and other resources such as office supplies, particularly those in lower-middle income settings.
- The resulting logic model demonstrates the need for multiple strategies acting through different mechanisms to address the determinants of implementation to improve care quality through a focus on child-centred outcomes.

## Background

Person-centredness is at the centre of holistic healthcare and a core commitment of the World Health Organisation [1–4]. In order to deliver child-centred paediatric healthcare, it is essential to understand what is important to children and their families [5, 6]. The United Nations Convention on the Rights of the Child emphasises the importance of children being involved in matters that affect them [7]. Patient-reported information is central to improving care and quality of life, and evidence demonstrates that children can reliably self-report [6, 8]. However, their voices have not always been prioritised in clinical care or research [9].

Person-Centred Outcome Measures (PCOMs) are standardised questionnaires used to assess patient (and sometimes family) outcomes of healthcare [10–12]. They are usually self-completed by the patient, or proxy-reported when a patient is unable to self-report [10–12]. Research demonstrates that PCOM use can improve care quality and patient outcomes [13, 14], support conversations about care, initiate decision-making through shared language, and empower patients and families [11, 15, 16]. Whilst PCOM use has become common place and the benefits recognised in adult healthcare, there is limited understanding of the impacts, benefits, and implementation of PCOMs in paediatric services [11, 17, 18].

Additional complexities must be taken into consideration in use of PCOMs with children as opposed to adults, such as the need for child-centred language and their varying cognitive and developmental abilities [8]. Prior reviews have not incorporated the three aspects of implementation, service-focused and clinical outcomes [19] (e.g. on acceptability or improvements in Health Related Quality-of-Life (HRQoL)) and tend not to be theory driven limiting rigour and translatability [11, 20–22]. Theoretically-informed implementation strategies are needed to implement PCOMs into routine paediatric practice for the benefit of children, their families, and health care services (Including health and social care professionals, hereafter “professionals”) [23].

This systematic review aimed to identify and appraise the evidence for implementation of PCOMs into paediatric healthcare settings and develop a logic model to identify potential strategies for implementation and their causal mechanisms. The review objectives were 1) to identify determinants and strategies for implementing PCOMs; 2) to describe the mechanisms through which barriers and facilitators to implementation interact to enable or hinder implementation of PCOMs; 3) synthesise the findings through the development of a logic model; 4) to appraise the quality of the evidence.

## Methods

This systematic review was conducted and reported in accordance with Preferred Reporting Items for Systematic Reviews and Meta-Analyses (PRISMA) guidelines [24]. The protocol for this review was registered on PROSPERO (International Prospective Register of Systematic Reviews; registration number CRD 42022330013).

### Searches

CINAHL, Embase, Medline, and PsycInfo were searched to ensure articles across medical, nursing, and psychological disciplines were considered [8, 25, 26]. Google Scholar was searched for additional articles or grey literature and references cited in selected articles were also searched [27]. Databases were searched from 2009 to present (25 March 2022) as 2009 was the year the patient-reported outcome measure programme was introduced into the NHS in the UK [28] as well as a shift in thinking about and focus on outcome measurement in health internationally [29].

Search terms were informed by child-focused research [8, 26] and search strategies from adult PCOM and implementation research [12, 15, 25]. Related Medical Subject Headings were also used in conjunction with the keywords based on the following concepts: *children* AND *outcome measures* AND *healthcare settings* AND *implementation*. Full search strategies for each database can be found in the supplementary files [S1, S2, S3 and S4].

### Study inclusion and exclusion criteria

#### Inclusion criteria

- Population: children ≤ 18 years old. Studies which include both children and adults were included if the data about those 18 and under are reported separately, or if the population were professionals working with children, or their parents.
- Intervention: Implementation or use of PCOMs or screening tools that are self-completed by a child in clinical care or proxy (parent/carer or professional) to improve care processes and/or outcomes.
- Outcome: data relating to barriers and facilitators to healthcare implementation and/or sustained use of a measure.
- Study types: Qualitative, case reports, quantitative (all experimental designs), mixed methods, service evaluations, quality improvement projects, audits. Systematic reviews were excluded but used for reference searching [27].

#### Exclusion criteria

- Population: Studies including only people aged > 18 years where they are not professionals working with or parents/carers of children ≤ 18 years old.
- Intervention: Studies where outcome measures are used to measure the effectiveness of an intervention or where measures are implemented into non-healthcare settings e.g., schools/social care
- Outcomes: data relating to scores, psychometric properties, or reporting symptom prevalence only
- Article type: Discussion/opinion articles, commentaries, editorials, letters, systematic reviews

#### Study selection

Articles identified in the search were imported to Covidence. HS screened titles and abstracts for eligibility; if there was not enough information to determine eligibility from initial screening, the full text article was screened. Full text articles were screened by HS and 10% were screened by a 2nd reviewer (DH). Discrepancies over eligibility of full text articles were discussed and resolved with a third reviewer (DB). Reason for exclusion of studies at the full text stage were recorded in a PRISMA flow chart [24].

### Potential effect modifiers and reasons for heterogeneity

Heterogeneity in the data is anticipated due to the inclusion of paediatric healthcare settings globally and across multiple health conditions, therefore the barriers and facilitators identified may be context specific.

### Study quality assessment

Study Quality Assessment was undertaken by HS. As multiple study types were included, several critical appraisal tools were used to assess the quality of studies of varying designs. The Critical Appraisal Skills Programme (CASP) tools [30] were used to assess study quality. Where there was not an appropriate CASP tool for the study design, the Joanna Briggs Institute (JBI) critical appraisal tools [31] were used. For mixed method studies, the Mixed Methods Appraisal Tool (MMAT) [32] was used. For quality improvement projects, the Quality Improvement Minimum Quality Criteria Set (QI-MQCS) [33] was used, and for non-randomised experimental studies of interventions, the Risk Of Bias In Non-randomized Studies – of Interventions (ROBINS-I) tool [34] was used. Articles were assessed against the items included in the checklists to develop understanding of the evidence rather than to exclude studies based on score. Study quality assessment results are presented in the results.

### Data extraction strategy

Data was extracted by HS. Data extracted in Covidence included: authors, title, date, country, aim, design and methods, sample (including: conditions and age of child, proxy inclusion, inclusion/exclusion criteria, sample size), healthcare setting, outcome measure used, administration data (how it is delivered and by who), implementation data (facilitators and barriers [12]), and patient outcomes data. Data were extracted from both results and discussion sections to capture investigators’ observations regarding implementation of the measure. Where data were extracted from the discussion section of papers, this was noted.

### Data synthesis and presentation

A narrative synthesis was conducted by HS to integrate qualitative and quantitative findings following the Guidance on the Conduct of Narrative Synthesis in Systematic reviews [35] with results discussed with RH, CES, DB. If disagreement occurred during these discussions, final adjudication (if needed) would be by RH. Preliminary synthesis involved tabulation, to develop initial descriptions of the studies and begin to identify patterns between studies. This was followed by a thematic analysis; deductively coding the extracted quantitative and qualitative data to the adapted-Consolidated Framework for Implementation Research (CFIR) constructs and sub-constructs [36, 37]. The adapted-CFIR comprises the original five domains from the CFIR with a sixth domain called ‘patient needs and resources’ [36, 37]. This gives person-centredness a greater focus to help ensure that patients’ needs are prioritised throughout all stages of the development, implementation, and evaluation of complex healthcare interventions [37]. This theory was selected as it is a well-established theory that has been evidenced to be effective for underpinning research and implementation of complex interventions in healthcare settings [23, 25, 40].

The effects of heterogeneity across studies were examined by comparing similarities and differences in outcomes across, study designs, settings, and populations to better understand the impact of context.

### Logic model development

The adapted-CFIR supported the data analysis and subsequent development of a logic model using Smith et al.’s [38] Implementation Research Logic Model template by HS but presented and discussed with members of the research team (RH, CES, DB). The determinants of implementation in the template map to the adapted-CFIR constructs and sub-constructs [36, 37]. This allowed thematically coded data to be mapped directly into the logic model as either determinant barriers or facilitating strategies.

## Results

### Review statistics

#### Search yield

**Fig. 1:**
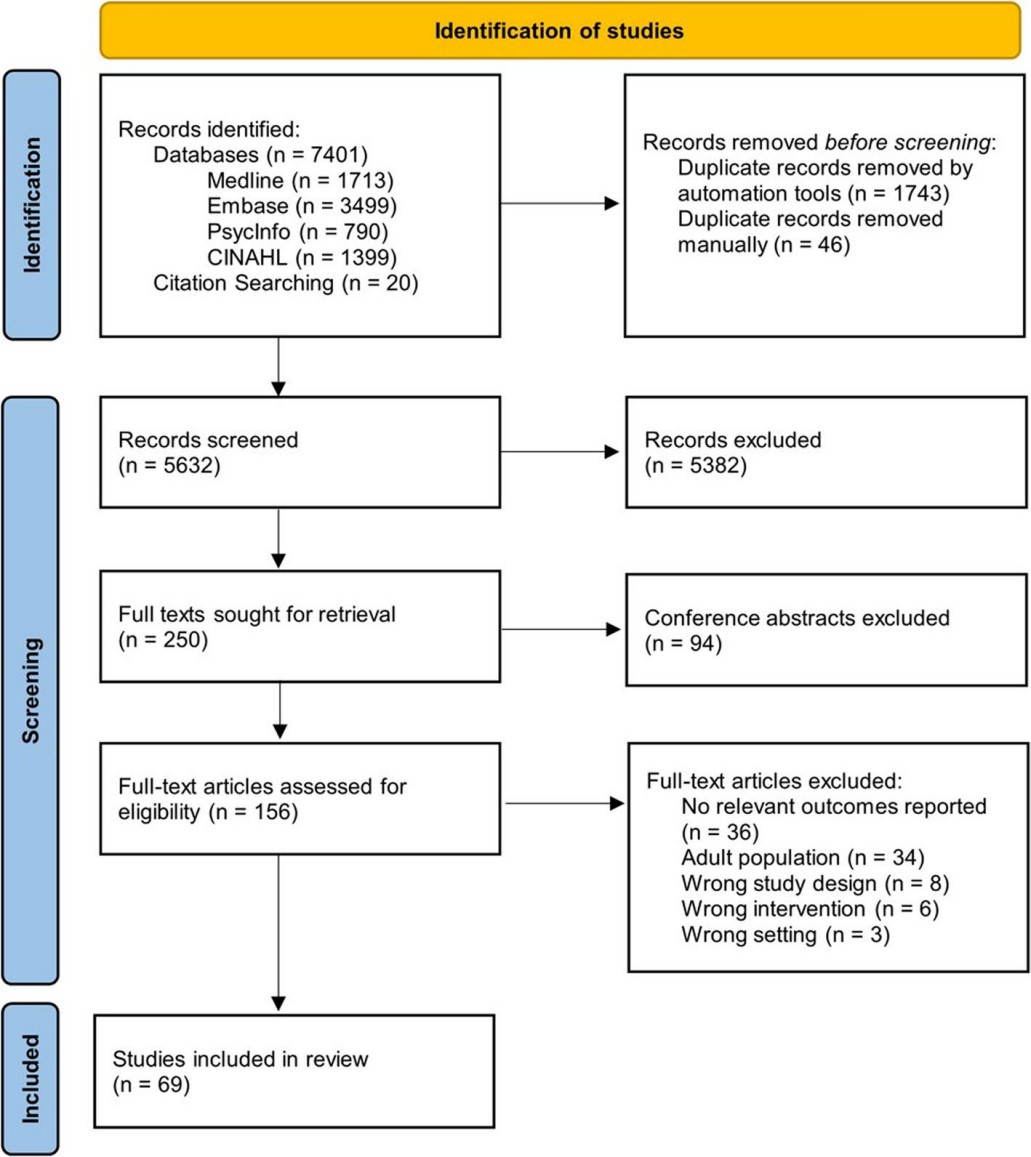
PRISMA flow diagram. Adapted from Page et al. (2021) [41]

The search yielded *N* = 7401 articles from databases and a further *n* = 20 from citation searches. After duplicates were removed [*n* = 1789], *n* = 5632 records were title and abstract screened, and *n* = 5382 were excluded. Of the remining *n* = 250 records, *n* = 94 were conference abstracts and thus excluded. Following full text review [*n* = 156], *n* = 87 were excluded (reasons: no relevant outcomes reported [*n* = 36], adult population [*n* = 34], wrong study design [*n* = 8], wrong intervention [*n* = 6], wrong setting [*n* = 3]), with *n* = 69 retained for the analysis. Figure 1 below shows a PRIMSA Flow Diagram of the inclusion/exclusion process and Table 1 summaries the included studies.

**Table 1:** Summary of characteristics of included studies

Author (date); Country; Study Design	Aim	Sample size (*N*); Setting; Age of Children; Condition(s)	Measure used; Completion method; How delivered	Main Findings
Anthony (2021); Canada; Qualitative [91]	To elicit perceptions from patients, caregivers, and professionals about the potential role of PROMs in the clinical care of paediatric transplant patients to inform effective implementation in this setting	*N* = *63*; Tertiary Hospital; 9–17 years; Organ Transplant	PedsQL4.0 Generic Core Scales/PedsQL4.0 Transplant Module; Completed on Dutch ePROM platform by patients and parents prior to appointment	Some adolescents did not want to share concerns about mental health and children did not always see the benefit. It was felt that bringing well-being into the clinical care conversation was a positive in terms of improving patient communication and engagement and informing practice. Ensuring the accurate interpretation of data was important to professionals and the need for a multi-disciplinary team to achieve this was highlighted both for interpretation and intervention
Barthel (2016); Germany; Mixed Methods [106]	To describe the implementation process of the Kids-CAT in clinical settings, focusing on the experience of children and adolescents regarding the user-friendliness and comprehensibility of the Kids-CAT and the experience of paediatricians with integrating the Kids-CAT Report into daily clinical routine	*N* = *32*; Tertiary Hospital; 7–17 years; asthma/diabetes/rheumatoid arthritis	Kids-CAT; study nurses supervised CYP while filling out the Kids-CAT in the clinic prior to consultation	Most participants felt the measure had a positive impact if patient-physician communication. Patient difficulties were better able to be identified and reports enhanced clinical patient data. Feasibility scores across perceived ease, need for help, and readability all indicated good feasibility. Paediatricians felt the measure would be desirable to integrate into routine practice and the computer-based application would facilitate this. Clinicians highlighted concerns when difficulties highlighted by the measure were outside their expertise or they lacked the resources to address them; multi-disciplinary joined-up working would better facilitate the use of the measure in practice
Batty (2013); UK; Mixed Methods [72]	To report on the implementation of Routine Outcome Measurement within three CAMHS affiliated to CAMHS Outcome Research Consortium	*N* = *127*; Secondary Care; children and adolescents unspecified; Mental Health	HoNOSCA/SDQ-parent/SDQ-teacher/SDQ-self/Conner' rating scale-teacher/Conners' rating scale-parent/C-GAS/CHI-ESQ/CAF; Measures included both self-report and parent/teacher proxy completion	Barriers to use included: lack of training, lack of awareness, lack of resources, and not valuing the measure. Completion of outcome measures was viewed by some as a ‘tick box exercise’ with little clinical or patient utility. Others felt that outcome measure were an important and useful way of recording progress. More information and training would better support the use of measures in practice as would integration with electronic patient records, and sufficient administrative support and resources Practical difficulties included low rates of completed questionnaires being returned. Some outcome measures also were not appropriate for some groups of children e.g., developmental difficulties, and often overlooked the impact of the child’s conditions on parents and carers
Bear (2021); UK; Cross Sectional [73]	To develop a self-report measure of practitioner attitudes to ROM in order to better understand the barriers to successful implementation in CAMHS	*N* = *184*; Secondary Care; children and adolescents unspecified; Mental Health	Standardized tools (e.g., SDQ)/Goals measures/ symptom tracking measures; Measures included both self-report and parent/teacher/ professional proxy completion	Professionals who frequently used outcome measures mostly felt service users were happy to complete them whereas those that used them infrequently, were less likely to endorse this. Professionals who frequently used outcome measures were more likely to describe them as helpful for planning support, deciding treatment approaches, and supporting shared descried making. Those in the frequent user group felt there was a strong evidence base for the use of outcome measures, were more likely to have attended external accredited training, and found training helpful compared to the infrequent user group
Berger-Jenkins (2019); USA; Quality Improvement [42]	To implement comprehensive screening for child behaviour and social determinants of health in an urban paediatric practice, and explore rates of referrals, and follow-up for positive screens	*N* = *349*; Primary Care; 6 months-10 years; healthy and medical/ developmental/behavioural/ psychiatric comorbidities	SWYC; parent-proxy completion only; both paper and electronic versions administered by clinic staff	Logistical/resource barriers included running out of photocopies of paper screens and staff unsure of eligibility for screening. There were also difficulties with integrating the electronic version of the measure into the electronic medical record. Other issues included families losing or forgetting to deliver completed screenings to providers. Education of front desk staff of the eligibility criteria increased screening rates as did including a reminder to return screens for families
Berry (2014); USA; Quality Improvement [43]	To increase knowledge, focusing on intent to change practice and implementation of routine early childhood developmental screening	*N* = *n/a*; Primary Care; 0–3 years; developmental delay	ASQ-3/ASQ-SE/PEDS/M-CHAT/EPDS/PSC/PHQ-9/HITS-Domestic Violence Questions/Addressing Mental Health Concerns in Primary Care: A Clinicians Toolkit; administered by professionals; parent-proxy completion only	A number of barriers to implementation and challenges to use in practice were identified including parental inability to complete screens due to language barriers, low literacy, and distraction of other children; high staff turnover; lack of an identified lead clinician (champion); not having sufficient resources or awareness of services professionals can refer to if children screen positive and both monetary and time costs of screening. Team meetings helped professionals better determine how to implement the screening process
Bhandari (2016); USA; Non-randomised experimental study [44]	To describe the first application of CHOIR in a paediatric pain clinic (Peds-CHOIR), with emphasis on the dual-tracking capacity for patient and caregiver reported outcomes	*N* = *352*; Tertiary Hospital; 8–17 years; pain	Peds-CHOIR (includes: Demographic and Pain History Questionnaire, Graphical body map; PCS-C/PCS-P, patient- and parent- reported PROMIS domains: mobility, pain interference, fatigue, peer relationships, anxiety, and depression); measures were administered through the CHOIR system; both child and parent-proxy completion were included	Clinical staff, patients and families all felt positively about the implementation of the Peds-CHOIR battery. Patients appreciated the ease of administration, short completion time, and visuals such as graph tracking of progress over time. When adherence dropped after 18 months, additional training and education for professionals in relation to its clinical utility and the benefits for patients facilitated an increase in use again. Completion by families at home before attending appointments improved clinic flow. Implementing and integrating Peds-CHOIR into clinic systems cost approximately $50,000, with $5,000- $7,000 estimated for annual maintenance
Bose (2021); USA; Quality Improvement [45]	To improve the identification and management of adolescent depression by implementing a practice-based, universal depression screening	*N* = *184*; Primary Care; 12–18 years; depression	PHQ-9 (Adolescents); implemented during annual wellness visits; completed by adolescents only	Implementation of the measure led to a statistically and clinically significant increase in diagnoses of depression and the percentage of adolescents who started treatment compared to pre-implementation
Brodar (2021); USA; Qualitative [46]	To describe paediatric diabetes care providers’ views on the relationship between psychosocial stress and diabetes, their experiences with psychosocial screening and psychological consultation within their clinic, and their suggestions for mental health professionals working with youth who have diabetes	*N* = *7*; Tertiary Hospital; 12–18 years; Diabetes	PHQ-A/GAD-7/eating disorders/diabetes stress/family conflict/ life satisfaction/ intrinsic motivation/ insulin adherence; routine screening for psychosocial concerns annually or more often during clinic consultations; adolescents only	A few professionals raised concerns around adolescents completing screens honestly and accurately, and there were some concerns around not having time to complete screens in busy clinics. Professionals felt the screen may help identify problems that would have previously not been identified in an efficient way and appreciated the inclusion of a diabetes specific measure. To better facilitate implementation and use in practice, professionals wanted more advance notice of which patients needed to be screened as well as more training and education related to the screening procedures. Screening information was perceived as positively impacting care, and further sharing of results with other team members was raised as something that would further improve care
Butz (2017); USA; Quality Improvement [47]	To demonstrate the capacity of a high-volume paediatric psychology outpatient clinic to reliably collect a quality-of-care measure	*N* = *n/a*; Secondary Care; paediatric unspecified; Psychological	PedsQL Core 4.0; parent proxy and CYP completion; provided by clinic staff and first and fourth visit	After training participating professionals, completion of the measure by families increased and by enlisting registration staff to identify missing forms, completion rates increased further. Automated alerts and reports for missing data supported further interventions to improve measurement collection and use of the process map suggested other areas of delegation by which supportive staff could aid clinicians in obtaining the data from families. Computerised administration facilitated the implementation of the measure. A shift in leadership led to a shift in focus away from the project
Campbell (2017); USA; Quality Improvement [49]	To assess changes in quality of care for children at risk for ASD due to process improvement and implementation of a digital screening form	*N* = *1205*; Primary Care; 16–30 months; Autism	M-CHAT / M-CHAT-R / M-CHAT-R/F; parent-proxy completion only; completed digitally on a tablet and screen report provided to clinician ahead of appointment	During the intervention 0% [n = 10] of clinicians felt using the M-CHAT disrupted workflow. Clinicians generally felt the M-CHAT added useful information to the clinical picture. 90% [n = 10] of clinicians felt that the digital M-CHAT-R/F improved autism assessment post intervention and 90% [n = 10] preferred the digital version and report to the paper version. Pre-intervention 88% [n = 16] clinicians felt the M-CHAT was easy for parents/caregivers and post-intervention this increased to 100% [= 10]
Campbell (2021); USA; Quality Improvement [48]	To increase the proportion of visits with screening for autism and the proportion of visits with referrals for autism evaluation	*N* = *n/a*; Primary Care/Community; 16–30 months; Autism	M-CHAT-R/POSI; parent-proxy only; delivered by professionals at well child visits	The proportion of referrals increased 1.5-fold in intervention clinics (from 1.3% to 3.3%) but not in community clinics Effective interventions included training the staff to administer a more sensitive screening instrument, prompting referral for scores suggestive of autism, adding reminders to the EHR, and adding autism evaluation in intervention clinics
Chen (2022); USA; Cross-sectional [50]	To characterize paediatricians’ perceived barriers and areas of confidence in assessing PROs in the U.S., and to test associations of these factors with implementing PRO assessment	*N* = *458*; Tertiary Hospital; Paediatrics unspecified; cardiology/endocrinology/ gastroenterology/haematology-oncology/nephrology/pulmonology/ rheumatology	N/A	Barriers included (n = 158) long length of PRO instruments (76.0%), limited skills on scoring PRO results (73.7%), limited ability to interpret PRO results (71.7%), Varying capabilities of children (66.8%). Only 26% of the paediatricians were confident in their ability to administer PRO instruments. 44% of paediatricians felt confident that PRO assessment provides more benefits to patients than relying on clinical judgement alone and 40% of the paediatricians indicated that PRO assessment is compatible with standard practice. Paediatricians in academic settings had more interest in assessing all PRO domains (except emotional and social well-beings) compared to those who worked in private settings (all p-values < 0.05). By paediatrician characteristics, those aged over 40 years reported more barriers to PRO assessment than aged 20–40 years
Corathers (2013); USA; Quality Improvement [51]	to evaluate the prevalence of depressive symptoms adolescents with type 1 diabetes, to quantify the number referrals generated from screening and to evaluate patient and staff acceptance of screening	*N* = *541*; Tertiary Hospital; 13–17 years; Type 1 diabetes	CDI; CYP completion only; CYP completed the screen in the examination room simultaneous to nursing intake	The majority (91.5%) of patients felt depression screening was important and all staff felt depression screening was highly important
Cox (2021); USA; Qualitative [52]	To understand the real-world barriers to PROMIS Paediatric clinical use as identified by clinicians and health system leaders	*N* = *18*; Tertiary Hospital; Paediatric unspecified; population unspecified	PROMIS measures; CYP and parent proxy-report	Concerns around wording of items and language barriers for non-English speaking CYP/parents and understanding of items were raised as potential issues. Inadequate privacy mechanisms or lack of communication about privacy mechanisms can result in patients/parents not completing measures. Engaging stakeholders, communicating the purpose of PROMIS measures to clinicians and patients, and training providers and other staff on how to administer, score, and interpret measures were identified as facilitators. There were logistical issues noted with integrating measures into EHR systems and the resources required for doing so meaning some health systems relied on pen-and-paper administration
Cunningham (2020); Canada; Non-randomised experimental study [92]	To present one example of implementation science in a preschool speech-language service system	*N* = *45*; Secondary Care; 0–6 years; speech and language	FOCUS/FOCUS-34; parent proxy completion only; administered by Speech-Language Pathologists at six-month intervals	Positive ratings increased across the survey items post webinar intervention relating to regular use of outcome measures, recognition of the evidence relating to the development and validation of the FOCUS, how to implement, use, score, and interpret FOCUS, the benefits and value FOCUS has
Cunningham (2018); USA; Quality Improvement [53]	To evaluate the presence of anxiety, pain, and functional disability in patients presenting with abdominal pain; replicate past research that revealed an association between anxiety, pain, and disability in a large clinical population; create a systematic approach to managing youth with FAPD on the basis of risk status; and quantify the number of psychological referrals generated after routine screening	*N* = *5221*; Primary Care; 9–18 years; functional abdominal pain disorders	SCARED child report/FDI-child version/The numeric rating scale for pain; Child self-report only; screens were administered during the pilot by paper and pencil by clinical staff, and then during larger scale implementation a web-based assessment process was conducted on a table provided at clinic check-in	Professionals felt screening revealed important information that would not have been identified in a standard visit, helped to frame the conversation with patients and was a positive experience that created a systematic approach to care and increased referral rates (After implementing the screening, psychological referrals rose from an average of 8.3 per 1000 patients per month to15.2 per 1000 patients per month). However, time, consistency, and limited resource access created barriers to implementation and use in practice
Davies (2021); South Africa; Non-randomised experimental study [101]	To demonstrate that mHealth technologies have the potential to improve the management of epilepsy in Africa	*N* = *40*; Tertiary Hospital; 4–16 years; Epilepsy	CHU9D/EQ-5D-Y/custom medication adherence, ketogenic diet, and sleep questionnaires; Child and parent proxy report; Aparito app on smart phone with linked wearable wrist worn device pushed notifications to prompt questionnaire completion every 30 days, and every day for a yes/no sleep question	There were a number of costs to patients including the cost of data for mobile app, phones, and repairs and for the institution costs relating to platform configuration, cloud hosting, personnel, devices and replacements. 87% of participants had to be given smartphones with better capabilities upon enrolling into the study as they either did not have smartphones or had phones that were not compatible with Bluetooth. Three phones were lost, three were stolen and ten stopped functioning. Nine participants had handsets re-paired or changed, but lost or stolen phones were not replaced
Edbrooke-Childs (2017); UK; Cross-sectional [74]	To examine the association between PROM, use and clinician demographic characteristics, attitudes and efficacy	*N* = 109; Secondary Care; children and adolescents unspecified; mental health	N/A	Mean scores for PROM use and PROM self-efficacy were higher for clinicians who reported having received training in the use of PROMs than for clinicians who reported not having received training. However, there was not a significant difference in PROM attitudes between clinicians who reported having received training and those who reported not having received training. Clinicians with more positive attitudes or self-efficacy regarding PROMs had higher levels of PROM use than clinicians with less positive attitudes or self-efficacy regarding PROMs
Eilander (2016); The Netherlands; Mixed Methods [82]	To assess whether monitoring QoL improves well-being and care satisfaction of adolescents with type 1 diabetes	*N* = *157*; Tertiary Hospital; Adolescents Unspecified; type 1 diabetes	DM-Y/ MY-Q; CYP and parent-proxy completion; measures were administered by professionals prior to consultation in clinic	All teams struggled with the logistics of DM-Y, including room to complete the MY-Q, requesting adolescents to come earlier for this purpose, problems with the web-based MY-Q, time to consult and discuss within the regular appointment. Motivations for use included: focus on the broader context of the child, recognition of interactions between physical and psychosocial factors, external motivations (guidelines), endorsement by external association, partnership between clinics, financial benefits, being able to objectify their clinical impression of QoL of their patients, usability. According to 75.0%, DM-Y did not interfere with regular medical care (19.4% neutral). Collaboration within teams with regard to DM-Y was considered good by 61.1%
Engelen (2010); The Netherlands; Case Report [18]	To provide a thorough description of the development and implementation of a PRO on HRQoL‚ the QLIC-ON PROfile, in clinical paediatric oncology practice	*N* = *84*; Tertiary Hospital; 0–18 years; cancer	QLIC-ON-PROfile: PedsQL/TAPQOL; included both child and parent-proxy completion; measures were completed electronically	Training programmes and engagement of clinicians in the development process supported implementation and use in practice. For CYP and families, explanations of what, why, and how to complete facilitated measure completion
Fäldt (2019); Sweden; Mixed Methods [99]	To investigate nurses, experiences and sense of competence when using the ITC communication screening at the 18-month health visit	*N* = *36*; primary care; 18 months; communication disorders	ITC; parent proxy completion only; administered by nurses at health visit	The length of the ITC as well as the language used were considered potential barriers, both in terms of the time it would take to complete, as nurses noted they often did not have time. For parents with reading difficulties, cognitive disorders, or those who were not proficient in Swedish, it would be difficult to understand. A low percentage of parents completed and brought the ITC to visits, and this was in part due to the check list having been implemented in the context of research. ITC led to more referrals and gave nurses an objective measure, making them feel more secure in the developmental check-ups
Fält (2020); Sweden; Mixed Methods [100]	To describe a facilitation programme developed to support the introduction of SDQ in clinical practice and evaluate how nurses perceived the facilitation strategies used	N = 68; Primary Care; 3–5 years; mental health	SDQ; parent and teacher proxy completion (only included data regarding parent proxy completion; Nurses administered SDQ to parents	More than half (58%) of the nurses reported of encountering negative reactions from parents in relation to the SDQ. Other issues with items included them not being age-appropriate or difficult to interpret, as well as language barriers when Swedish was not their first language. Nurses felt the SDQ improved quality of the check-ups, providing more structure, and a basis for discussion with parents (particularly for discussing sensitive topics) and increased focus on the child, as well as increasing their knowledge of the child’s mental health more broadly. At the end of the trial, 96% stated it was now an integral part of routine practice. Nurses appreciated the facilitation strategies. In the survey, educational meetings and educational outreach visits received ‘very important’ scorings, at 41% and 33%, respectively
Fein (2010); USA; Non-randomised experimental study [54]	To determine the adoption rate of the Web-based BHS-ED system during routine clinical practice in a paediatric ED, and to assess this system’s effect on identification and assessment of psychiatric problems	N = 857; Tertiary Hospital; 14–18 years; mental health	BHS-ED; CYP completion only; emergency department nurses or technicians asked patients to complete a web-based screen in private where possible after patient medical assessment	After implementing the screen, there was a significant increase in identification of patients with psychiatric illness (2.5% to 4.2%) indicating that even when not all patients can be screened, the implementation of this process can increase the likelihood of discovering psychiatric illnesses in the emergency department
Fenikilé (2015); USA; Qualitative [55]	To explore potential barriers to adoption of recommended screening for autism by family physicians at 18- and 24-month well-child visits	*N* = *15*; Primary Care/Community; 18–24 months; autism	Autism screening tools (M-CHAT); parent proxy completion only; administration of screen in well-child care visits	In general, participants who have been in practice longer did not agree with the American Academy of Paediatrics’ universal screening recommendation for a specific condition. Some participants indicated that they were not aware of any Association guideline recommending routine screening for autism. Some participants viewed screening for a specific condition to be an inefficient use of a physician’s time due to time constraints of clinic visits, and lack of resources
Friedel (2020); Belgium; Qualitative [105]	To assess the face and content validity, acceptability and feasibility of a French version of the CPOS	*N* = *44*; Tertiary Hospital/Community; 9–18 years; Life-limiting and life-threatening conditions	C-POS/adapted C-POS/ SEIQoL/QOLLTI-F; both CYP and parent proxy completion	CPOS was perceived as a useful tool that shined a ‘warning light’ on overlooked domains, helping professionals to individualize and to improve the care provided. Parents and children all expressed positive feelings about the interviews. Furthermore, parents said that it allowed them to identify what helped them and to express to one another their mutual sense of gratitude
Fullerton (2018); UK; Mixed Methods [75]	To examine the impact of training supervisors in using PROMs on clinical practice, given the importance of leadership when changing behaviour	*N* = *50*; Secondary care; children unspecified; mental health	N/A	Supervisors had more positive attitudes to administering PROMs and using feedback from PROMs and had higher levels of self-efficacy about using PROMs in supervision, after UPROMISE training. Supervisees commented that supervisors used PROMs to a greater extent after training. Supervisees commented that supervisors were more confident in their use of PROMs in supervision
Gerhardt (2018); USA; Case Report [56]	To describe the development and implementation of a PRO program at Cincinnati Children’s Hospital Medical Centre that can serve as a standardized approach for the use of PROs in a clinical setting	*N* = *n/a*; Tertiary Hospital; children unspecified; multiple condition groups including: asthma, allergy, Autism, diabetes, anxiety, cardiology, nephrology, depression	26 Generic PRO Instruments (covering emotional health, behavioural health, social health, physical health, overall health (PedsQL), and care management)/42 disease-specific PRO instruments; child self-report and parent-proxy measures included	Components identified as essential to successful PRO implementation: Commitment (Identification of a committed clinical team leader and team)‚ Instrument Selection (Selection of an instrument that addresses the identified outcome of interest), Scores (specification of threshold scores that indicate when an intervention is needed), Interventions (identification of clinical interventions to be triggered by threshold scores), Training (training for providers and staff involved in the PRO implementation process—Trained staff ensures continued use), Reliability and Use (Measurement and monitoring for PRO reliability and use—Too many questions is too burdensome)
Godoy (2021); USA; Qualitative Research [57]	To describe barriers to, and facilitators of, universal MH screening implementation, the perceived impact of such screening, impressions of a screening-focused QI Learning Collaborative, and lessons learned	*N* = *11*; Primary Care; 3 moths-18 years; mental health	ASQ:SE/SDQ/PHQ-9; child and parent proxy completion; administered by PCCs in well-child visits	Provider capacities/characteristics that facilitated implementation included factors such as PCC knowledge, understanding, attitudes, and confidence related to identifying and addressing mental health (MH) issues. Patient capacities/characteristics that acted as barriers to successful screening and referral included language, literacy, and parent motivation, previous child and family experiences of mental health issues, and the family’s ability to navigate resources
Harding (2019); UK; Qualitative [76]	To elicit views on the domains/items to include in a PCOM, implementation challenges and requirements for use in routine care by practitioners	*N* = *36*; Tertiary Hospital/Community; paediatric unspecified; Life Limiting Conditions/Life Threatening Illness	N/A	Measures should be able to be used by CYP with a wide range of cognitive abilities and also demonstrate proxy validity and responsiveness. Measures should be child friendly, engaging and brief and applicable throughout the child’s life and into end-of-life phases of illness. There should be appropriate mechanism developed to allow results to be shared across multiple agencies and services involved in care but there must be clarity on who would have access to data with clear guidelines on storage, access and use of the data. The perception of the tool is key to implementation: it must not be seen as a ‘test’ of the quality of informal parent care provision nor raise unrealistic expectations of care. The purpose of outcome measurement should be clearly aligned to improving person-centred care. professionals should be trained in how to interpret results at an individual and population level
Hardy (2015); UK; Non-randomised experimental study [77]	To introduce a screening service that would provide earlier identification of the social and emotional difficulties of CiC aged under 5 years in a 12-month period and to gain a greater understanding of the level and type of needs among this population	*N* = *63*; Secondary Care/Community; 6 weeks-65 months; social/emotional wellbeing	SEGC/ASQ-SE/PCIS; parent/carer proxy completion only; administered by community paediatrician at initial health assessment home visit for children in care	Independent reviewing officers, social workers, and foster carers provided feedback on the screen out of 5 (5 being a great deal). In terms of the screen adding understanding of the child’s needs the mean scores were 4, 3.7, and 3.5 respectively. In relation to the screen contributing to care planning the mean score from independent reviewing officers was 4.3 and the mean score for social workers was 3.3. The implementation of the screening increased the proportion of children identified with difficulties significantly
Haverman (2014); The Netherlands; Case report [83]	To provide a thorough description of the implementation of ePROs in daily paediatric clinical practice in line with the methodological recommendations and decisions described in the International Society for Quality-of-Life Research guidelines	*N* = *n/a*; multiple settings; 0–18 years; 17 paediatric patient groups including: rheumatology, nephrology, coagulation disorders, HIV, cystic fibrosis, and oncology	KLIK eProfile (TAPQOL/ PedsQL Generic Core Scale); CYP and parent-proxy report; parents/patients are invited to register on the website, before the consultation with the paediatrician, Patients without home Internet access or who do not complete the questionnaires prior are given the opportunity to complete the questionnaires at the clinic	The biggest issue was clinicians forgetting to discuss results with patients. Clinicians felt the KILK adds value and patients, and parents recognise this and that graphs representing score changes overtime were beneficial. The web security and patient privacy of the platform facilitated its used as did tools to support scoring and training for clinicians
Haverman (2013); The Netherlands; Cohort Study [84]	To investigate the effectiveness of ePROs in clinical paediatric rheumatology care	*N* = *176*; Tertiary Hospital; 0–18 years; juvenile idiopathic arthritis	KLIK (TAPQOL/ PedsQL Generic Core Scale/ PedsQL parent report/ The Dutch CHAQ/ 100-mm VAS for the evaluation of pain and overall well-being/ DISABKIDS arthritis module; CYP and parents were provided login details for the KLIK website to self-complete the PROMs ahead of clinic appointments	Overall, the evaluation of the use of the ePROs was positive. In 88% (first intervention) and 80% (second intervention) of the consultations, the parents regarded the ePROfile as useful. They regarded the ePROfile as helpful for themselves and as helpful for their child. In 94% of the first intervention consultations and in 91% of the second intervention consultations parents felt the PROfile reflected their child’s HRQoL adequately. The Paediatric Rheumatologists reported that they were more satisfied with the provided care during the consultations in the domains of emotional support for parents and child, meeting the needs of the child. CYP reported that discussing the ePROfile with the clinician was ‘normal’
Herbert (2019); USA; Mixed Methods [58]	To evaluate the acceptability and usefulness of brief mental health screening during paediatric subspecialty clinic visits	*N* = *523*; Tertiary Hospital; 5–17 years; mental health in allergy/immunology/haematology	PROMIS profile; child and parent proxy completion; administered on iPads in clinic waiting rooms before appointments	Most of the professional interviews (n = 67; 87%) indicated that medical providers referred to the PROMIS Patient Summary to guide at least part of the visit. Most parents agreed or strongly agreed that it was easy to fill out on the iPad, the length was appropriate, and the questions asked were appropriate for the care of their child. However, some parent noted difficulty completing the PROMIS Paediatric Profile in the waiting room and/or concern that it was not used by their clinician
Hinds (2013); USA; Cross Sectional [59]	To assess the ability of children and adolescents with cancer to complete the PROMIS paediatric measures electronically and to establish preliminary validity estimates of the PROMIS paediatric measures in paediatric oncology	*N* = *200*; Tertiary Hospitals; 9–17 years; cancer	Eight PROMIS paediatric measures (Physical Functioning, Mobility, Physical Functioning, Upper Extremity, Pain Interference, Fatigue, Depression, Anxiety, Peer Relationships, and Anger); child completion; electronically administered using laptops or computers available in the clinical settings	There was generally little difficulty with completion, however there were three cases where parents had some difficulty manipulating the computer screens and two cases of children under 11 years old where one took an unusually long time to complete the measure and the other required assistance to stay focused
Jonsdottir (2020); Iceland; Mixed Methods [107]	To study the implementation of an early detection program for ASD within well-childcare in PHCs and to evaluate its initial outcome	*N* = *1596*; Primary Care; 30 months; Autism	M-CHAT-R/F; parent proxy completion only; Administered during well-child visits	All the nurses expressed having positive experiences and expressed a positive attitude towards the adoption of universal screening for Autism, and there was an interest in doing so at both the 18- and 30-month well- child visits. Although there was also an interest in training, there were concerns as to the extra time this would require
Kazak (2017); USA; Qualitative [60]	To identify how multidisciplinary paediatric oncology health-care providers perceive psychosocial risk screening to identify factors in uptake and implementation	*N* = *15*; Tertiary Hospital; children unspecified; cancer	PAT; CYP and parent-proxy completion; initial screening using the PAT was conducted between 24 h after diagnosis to up to 1 month later, usually administered on a tablet computer	Successful implementation requires planning including determining who should administer the screener as well as how results can be effectively communicated to key health-care team members. professionals must recognise, understand, and appreciate the importance of screeners (’buy-in’ or ‘ownership’). Accessible training resources to facilitate the use of the PAT, including guidance on scoring, interpretation, and clinical care were seen as valuable next steps that could further enable broader implementation. Engaging families in a process of screening, characterized by relationships of trust, was identified as an important requisite as well as its acceptability to families. Time to complete screening, from the perspective of both the family and staff, is a challenge. Concern was expressed about Survey fatigue, and the logistics of families completing a screener in a busy clinic, as well as language and literacy barriers where families would require additional staff support to complete the screen
Kazak (2019); USA; Non-randomised experimental study [61]	To facilitate implementation of the PAT in English and Spanish in oncology in three states in the South-eastern United States	*N* = *16*; Tertiary Hospital; paediatric unspecified; Cancer/Stem Cell and Organ Transplant/ Histiocytosis/ Sickle Cell Disease/Cardiac Disease	PAT; child and parent proxy completion; measure was administered by a clinician and then reviewed by a social worker or psychologist	Most programs (78%) indicated that the PAT was very or extremely useful in their clinical work. With respect to using the PAT results to guide intervention, more than half of the programs indicated that they always used it to guide intervention and most used it at least some of the time to do so. Participants were very positive about potential benefits of using the PAT. Both before and after the workshop professionals felt that PAT implementation would facilitate communication among staff and with families. They also all indicated that PAT would facilitate clinical care, deliver care efficiently, and promote positive medical and psychosocial outcomes. Open text comments show how participants viewed the PAT as an asset to their clinical work. The most common expected challenges were related to reimbursement, technical issues, and integrating the results in the EHR. At post-implementation challenges were reported related to support from the medical team and champions
Kendall (2019); UK; Qualitative [78]	To evaluate the acceptability and understanding of the ASQ-3 in England by health professionals and parents	*N* = *125*; Community Services; 2–2.5 years; Healthy/Developmental Delays	ASQ-3; parent proxy completion only; administered to parent by community professionals	Parents and HPs were equally positive about the opportunity to work in partnership in relation to the child's development. In general, most parents and HPs accepted the ASQ-3 as a measure that provides useful information about a child's development at 2 years. However, some parents indicated that they had been worried before or during completion of the ASQ-3 and perceived it as a’test’ and worried that their child might ‘fail’; regularly ticking ‘not yet’ caused most anxiety. The language was also considered very American. There was wide variation, both across and within sites, in how the ASQ-3 was being used in part due to how it was introduced conceptually to HPs (at management level)
Kip (2022); Malawi; Qualitative [108]	To assess barriers and facilitators to implementing HEADSS for adolescents with HIV attending Teen Club Program in four selected health facilities in Malawi	*N* = *20*; Community Services; adolescents unspecified; HIV/AIDS	HEADSS; CYP completion only; administered by professionals in Teen Club clinics	The participants acknowledged that this psychosocial screening tool can guide better systematic counselling, build better client provider relationship, improve quality of care, and be good for holistic psychological assessment of ALHIV. Many of the participants further indicated that the HEADSS screening could fit into their existing work practice and was described as not very complex to implement. The majority had some reservations because the tool was not culturally specific to Malawi context, which was viewed as a barrier for the implementation. Participants also indicated that the screening tool was in English and could not easily translate the contents into the local language. Some of the participants believed that ALHIV will be suspicious that the HCPs are policing on them when they ask them questions. HCPs might be willing to implement HEADSS screening if they were given some incentives in a form of cash
Krishna (2019); USA; Quality Improvement [62]	To implement computerized diagnostic and history assessments for outpatient mental health visits in the ambulatory psychiatric clinic of a large paediatric health system	*N* = *1489*; Secondary Care; paediatric unspecified; psychiatric	Diagnostic screener/ Computerized assessments; administered to patients on iPads before appointments	Provider response was highly positive. Average provider response on the 5-point Likert scale was 4.36 (5 = strongly agree 3 = neutral). 67% of the providers believed that the data changed how they approached their diagnostic visits. 78% of providers indicated that the assessment improved the efficiency of their visits. On average, they reported that 16 min of time has been saved of a 90-min assessment. Providers overwhelmingly reported that this extra time was utilized for improved patient care. Providers rated it as easy to use, with useful and relevant questions. A core team and champions team served a support function, passing on their experience with the implementation process to other clinics, providers, and staff
Kwok (2022); Canada; Qualitative [93]	To investigate commonly experienced facilitators of and barriers to implementing the FOCUS in clinical practice from the perspectives of SLPs	*N* = *37*; Secondary Care; 0–5 years; Communication Disorders	FOCUS; parent proxy report only; delivered by Speech-Language Pathologists in the PSL programme (mandated by programme)	Barriers included: integrating FOCUS into already busy sessions, incompatible schedule between FOCUS and clinic visits, workload burden due to complex and redundant steps, FOCUS data did not impact clinical practice, FOCUS data were not used to make system-level decisions, FOCUS data were not valid, damaging rapport with family, forgetting to administer the FOCUS, not feeling confident in answering specific questions, uncertain how to interpret and explain items on the FOCUS, not knowing administrative schedule, FOCUS contradicted professional roles, negative emotions, lack of optimism. Domains where more facilitators than barriers to implementation were identified included optimism, intentions, behavioural regulation. Facilitating factors included: creating a reminder system, keeping resources available in session, adjusting as needed, personnel support, technology support, internalised intention, FOCUS as a tool to gather parents' perspectives, not an 'onerous' task, developing skills, associating FOCUS with an assessment, awareness of importance
Lalloo (2014); Canada; Qualitative [94]	To assess the clinical feasibility of the PQ from the perspective of adolescents with chronic pain and members of their interdisciplinary paediatric health team in the context of a follow-up chronic pain clinic appointment	*N* = *25*; Tertiary Hospital; 12–18 years; Chronic Pain	PQ; CYP completion only; CYP completed the PQ and clinic comparator tool on a laptop computer in a quiet study rom in clinic ahead of their scheduled appointment	Adolescents described the PQ as useful for initiating and promoting clear communication with the health team and providing a more complete understanding of pain experience. 4 adolescents (24%) referred to a sense of ownership and control over creating their own pain record and described using it as easy or very easy. Given a choice of methods for communicating their pain in the clinic, 15 (88%) adolescents preferred the PQ. Professionals also noted the ease of interpreting the adolescent-generated PQ pain records. The transferability of the PQ software across different web platforms was seen as beneficial. Ensuring patients privacy as they completed the tool was a challenge; technology requirements (Internet-connected computer; printer if hard copies are desired), adjusting workflow to accommodate patient completion and team interpretation of the PQ were also potential barriers to implementation and use
Lynch-Jordan (2010); USA; Quality Improvement [63]	To illustrate the process of using improvement science methodology to put into practice an efficient, clinically useful measurement tool to evaluate patient functional status before, during, and at the conclusion of treatment among children and adolescents referred for outpatient, behavioural. pain management	*N* = *107*; Tertiary hospital; M = 15.2 years; Chronic Pain	FDI; CYP completion; psychologists administered the FDI to every patient referred for pain in the session or it was self-administered in the waiting room	The biggest barrier to regular FDI administration was clinician forgetfulness (96.5%) related to time constraints (i.e., patients arriving late) and excessive paperwork (i.e., during the initial evaluation). Graphical representation of data in charts were viewed by psychologists as logical, compelling markers of patient progress. The addition of this component to the treatment session was not viewed as prohibitive in terms of efficiency or effort. Psychologists denied any adverse reactions by patients. Psychologists provided qualitative feedback that described the FDI as easy to administer and score; non-disruptive to the flow of the treatment session; and valuable in providing a quick measure of functional status to the psychologist and patient. It served both as an indicator of patient progress but also reportedly enabled psychologists to clearly identify areas of deficits (e.g., physical activity and sleep) that could be targeted for specific intervention in treatment. Psychologists’ observations were that patients became encouraged if their scores/run charts visibly dropped (indicating less functional disability), and they frequently remembered their scores from previous sessions without clinician prompting
Mansour (2020); USA; Quality Improvement [64]	To implement a standardised questionnaire to improve screening for depression by 60% in adolescents from 12 to 17 years of age	*N* = *109*; Primary Care; 12–17 years; depression	PHQ-A; CYP self-report only; administered by professionals in session	By the end of the study period, screening rate had increased from 0 to 70%. Lack of resident education causing poor adherence was identified as a barrier. Sending emails to residents with instructions on how to use the depression screening questionnaire increased screening rate from 0 to 31%. Microsoft PowerPoint was used to create educational lectures regarding depression screening, and the need for implementing the PHQ-A was high-lighted. Literature supporting the use of a standardised tool was reviewed during the presentation—screening rate increased from 31 to 81%. Language barriers with patients was identified as a challenge to adherence. Highlighting patients between 12 and 17 years of age on the printed patient schedule for each resident daily was a visual reminder for adolescents to be screened for depression led to the screening rate increasing. Residents were encouraged to continue using the screening tool even after the conclusion of the QI study
McCarthy (2016); Australia; Mixed Methods [103]	To investigate the feasibility of administering the PAT2.0 psychosocial screener to parents following their child’s cancer diagnosis and to examine oncology health-care professionals (HCPs) perspectives on the of the PAT2.0 screening tool in their clinical setting	*N* = *162*; 0–18 years; Tertiary Hospital; Cancer/cancer-related haematological disease	PAT2.0; parent proxy completion only; Clinical social workers approach families following diagnosis to complete the PAT2.0 and return it directly to their social worker or in a sealed envelope to outpatient or inpatient administrative staff	Eighty-five percent (n = 87) of parents reported they had no concerns about the PAT2.0 being stored in their child’s medical chart. The overall return response rate of 83.25% indicates that the PAT2.0 was acceptable to the majority of families. The majority of parents reported that the PAT2.0 was easy (82.0%) or somewhat easy (18.3%) to complete. Of the HCPs individually interviewed, 53.06% felt the communication summary provided them with new information and 69.64% reported this was useful in providing clinical services to families (i.e., raised issues that would otherwise not be detected, opened communication about the family’s concerns). However, only a minority (22.81%) of HCPs felt that the information received from the PAT2.0 impacted their clinical decision-making. Social workers also reported several perceived barriers to administering the PAT2.0, including delay in children receiving a cancer diagnosis, delay in families returning the completed PAT2.0, and additional workload. In particular, social workers commonly reported having to follow up with families sometimes several times to obtain the PAT2.0
Meryk (2021); Austria; Cohort Study [104]	To evaluate the feasibility and value of daily patient-reported outcome measures (PROMs) by children receiving chemotherapy for cancer	*N* = *12*; Tertiary Hospital; 6–18 years; Cancer	ePROtect patient portal (multiple symptoms PROMS including. pain, appetite loss/nausea, physical functioning, sleep quality); CYP completion only; Patients were instructed to complete the symptom monitoring once per day during the study period on a mobile device or tablet and, patients were reminded during inpatient treatment to complete the questionnaire each day before the morning round	Children rated the PROMs as useful and easy to use and gave a high rating for satisfaction
Orava (2019); Canada; Mixed Methods [95]	To evaluate the implementation supports and adoption of the Chronic Pain Assessment Toolbox for Children with Disabilities (the Toolbox) to enhance pain screening and assessment practices within a paediatric rehabilitation and complex continuing care hospital	*N* = *224*; Tertiary Hospital; children unspecified; Chronic pain/Cerebral palsy	Body diagram/CALI/ PPP/PPIS; CYP and parent proxy completion; routine screening for pain on admission or during a visit with a professional, followed by an assessment using a systematic approach and validated tools	Implementation of the Toolbox led to an increased in the number of CYP who had a conversation about pain (presence/absence of pain) recorded. Advantages of the tools included being able to gather information about a pain history; objectively measuring the site, source, and ways in which chronic pain interfered with daily living activities; and helping clients, families and HCPs describe different instances of pain (such as during cramping, tightness, and dystonia); as well as promoting joined up working, and communication/information sharing. professionals reported Toolbox was thorough and helpful, particularly clinical practice points and tools coring resources, visual prompts also helped to remind professionals to use it. Challenges reported included: difficulty using PROMs, paper copies not being available, and finding time to complete in busy clinic
Purbeck (2020); USA; Mixed Methods [65]	To examine the acceptability, appropriateness, adoptability, and feasibility of an MBC effort, the CIMI, across several child-serving settings (e.g., community mental health centre, residential treatment facility)	*N* = *70*; Secondary Care/Community; children unspecified; trauma/mental health	CIMI; CYP and parent proxy; used by clinic staff in consultations with children and families	Characteristics that facilitated implementation included the support of external change agents (implementation purveyors who were helpful in providing advice and sharing resources), formally appointed internal implementation leaders (supported staff becoming more comfortable using assessment battery), and CIMI champions (who kept staff on task and was supportive). For some, the assessment battery felt too long or intense. The availability of measures was also challenging with some noting that not all versions were available electronically and had to be done on paper with results manually entered. Complexity and design quality and packaging (e.g., structure and layout of the technology) made implementation challenging for some sites there were accessibility issues within sites whereby not all staff were able to access the CIMI. Generally, CIMI was considered acceptable, appropriate and feasible although staff did not fully feel that the technology enhanced their work
Robertson (2020); UK; Mixed Methods [79]	To investigate ophthalmic clinicians’ prior experience of, and future training needs for, using PROMs and their views about the barriers and enablers to future implementation in paediatric ophthalmology practice	*N* = *45*; Tertiary hospital; children unspecified; ophthalmology	VQoL_CYP/FVQ_CYP; child self-report	Only 22.2% had experience of using PROMs. PROMs were considered useful for detecting problems and concerns clinical assessments may not identify; making decisions; monitoring condition and response to treatment; and improve communication and joint decision making with patients and families. However, clinicians lacked confidence in explaining what scores mean or how they would be used
Santana (2015); The Netherlands; Non-randomised experimental study [85]	To describe the development and implementation of three programs for training clinicians to effectively use PRO data in routine practice	*N* = *n/a*; Tertiary hospital; children unspecified; population unspecified	KLIK PROMs; CYP and parent proxy reporting; administered electronically through KLIK portal	1-h group training with a theoretical and practical parts, including video material & a training manual was created to enhance effective use of the PROfile in clinical practice. Professionals sometimes forget to discuss the ePROfile. Professionals were positive about the use of KLIK and recognize the value added. They felt that parents and patients do not mind completing the questionnaires and benefit from using KLIK. The motivation of the multidisciplinary team was an important factor for this success, plus targeted initial support by the KLIK team. Patients were given direct feedback after they completed the questionnaires, helping them to understand the goal and motivating them to complete the questionnaires again
Schepers (2017a); The Netherlands; Cohort Study [87]	To determine the feasibility of the use of the ePAT in Dutch clinical practice approximately 1-month post-diagnosis, to evaluate the usability of the PAT ePROfile, and to determine possible differences in feasibility and usability for families with a universal versus an elevated (targeted or a clinical) risk score	*N* = *75*; Tertiary Hospital; 0–18 years; Cancer	ePAT/PAT 2.0; parent-proxy completion only; families registered online at the KLIK website, one parent per family completed the ePAT preferably within 1-month post-diagnosis	The following reasons were indicated by families that did not want to participate: too much effort; too many other things on our mind; no desire to complete extra questionnaires; we do not want to commit to anything extra than just the standard treatment. Reasons declared by the psychosocial team for not reviewing or discussing the PAT ePROfile results were as follows: logistics (i.e., too busy and/or no team meeting), psychosocial team members did not think it was necessary to discuss results because of a universal PAT score, no additional information derived from the PAT that had to be shared with the team, and that the family was not known to anyone from the psychosocial team
Schepers (2017b); The Netherlands; Mixed Methods [86]	To determine the of the KLIK method as implemented in outpatient paediatric cancer care and to study health care professional (HCP) reported barriers and facilitators for implementation	*N* = *233*; Tertiary Hospital; 0–18 years; Cancer	KLIK PROM (generic HRQoL questionnaires/ PedsQL 3.0 Acute Cancer Module); CYP and parent proxy completion; administered electronically on the KLIK online system	The reported facilitating factors were as follows: HRQoL problems were efficiently detected using KLIK method (87%), social support from KLIK coordinators (83%), normative beliefs around expectations of use (96%), simplicity of the KLIK method (86%), having sufficient knowledge to use it as intended (86%), 100% considered the opinions of patients/parents regarding the use of KLIK PROM as important and this was a motivating factor. HCP reported barriers were: social support (25% did not receive support), descriptive norms (25% indicated only a minority of their colleges actually used the KLIK method), 39% did not consider the opinions of the management team important thus reducing motivation to comply. Organizational (hospital) barriers were as follows: lack of formal ratification by managers, no replacement when staff left, lack of time available, unsettled organisation/organisational changes, lack of feedback to professionals about the implementation progress. Another perceived barrier to the intervention (KLIK method) was compatibility (24% of HCPs indicated that the KLIK method did not fit well with current routines)
Schreiber (2015); USA; Case Report [66]	To describe the use of a KT program to improve the knowledge and frequency of use of standardized outcome measures by paediatric physical therapists practicing in an outpatient clinic	*N* = *17*; Secondary Care; 0–18 years; conditions unspecified	GMFM-66/GMFM- 88; GMFCS and motor curves/PEDI TUG/TUDS/ 30-s walk test; administered to patients/families by physical therapists in clinic	The knowledge Translation programme increased professionals’ knowledge of test selection, administration, interpretation, and sharing of results
Schulte (2019); Canada; Non-randomised experimental study [96]	To implement standardized screening tools in a busy clinic setting; to assess the feasibility of administering these tools based on recruitment rates and acceptability; and to evaluate the psychometric criteria of these tools (i.e., construct validity, test–retest reliability and discriminative validity) at each stage of the cancer continuum (on treatment, off treatment)	*N* = *190*; Tertiary Hospital; 1–18 years; cancer	DT/PATrev/PedsQL generic core 4.0; CYP and parent proxy completion; on-treatment patients were approached by their social workers or a research assistant during in-patient stays or at clinic appointments/off-treatment patients were approached by research assistants during clinic appointments	The acceptability of the DT was rated significantly better by families off treatment compared to families who were on treatment
Sharples (2017); UK; Qualitative [80]	To explore clinician attitudes to outcome measures and, in particular, the facilitators and barriers to implementing outcome measures	*N* = *9*; Secondary Care; Children and young people unspecified; Mental health	Unspecified outcome measures; CYP completed; clinician administered in sessions	Barriers included resources (in terms of information systems, administrative processes and time within sessions), clinicians and service users struggling to use measures when they were not seen as appropriate, and the structured content of measures as resulting in them, at times, being misinterpreted by service users, or causing distress and disengagement. Facilitating factors included training in and practical experience of using outcome measures with ongoing support needed to sustain use, recommendations to support use in the future at service level, and the structured content of measures was also described as being useful to service users to help frame discussions of presenting problems and treatment planning
Silver (2017); USA; Qualitative [67]	To describe the dynamic processes that support and/or hinder the implementation of early childhood screening by analysing prospective, longitudinal, qualitative data from a grant-funded project that integrated early childhood screening within two urban paediatric primary care clinics serving high-risk families	*N* = *63*; Primary Care; 0–8 years; mental health	CWS (ASQ-3, ASQ-SE; ECSA, PEDS, PSC); parent-proxy only; CWS-eligible children and families were approached upon clinic entry and completed CWS tools and summary forms were filed in the child’s medical record	Both Pediatrics and Implementation stakeholders mentioned improved coordination over time, because of increased communication, more positive interactions and. relationships, and integrating the screenings into the EMR (at one site) to support collaboration. Being able to scan in and add screen to EMR was helpful but there were barriers to integrating. Being able to bill for screens also to supported sustainability. However, there were concerns about family’s perceptions of being asked to complete CWS (e.g., stigma), and the reading level required to complete it being too high. Staff also struggled with having enough time to complete it, score it, and feed it back to families in visits, clinicians found it difficult to interpret results. Low physician buy-in of CWS and their ownership over the screening process was a barrier that remained even with increased positive perceptions of the screening overtime, raising concerns about sustainability of the intervention. Limited resources, in terms of space, computers and staff was a barrier. Funding was also noted as a necessity for sustainability
Stinson (2012); North America; Mixed Methods [110]	To develop and test the feasibility of SUPER-KIDZ	*N* = *204*; Tertiary Hospital; 4–18 years; pain/rheumatology	SUPER-KIDZ; CYP and parent-proxy completion; measures were administered by research assistants prior to rheumatology appointments on paper, and electronically on handheld devices and on laptop computers	There was no difference in the overall preferred medium for youth or parents. There was however a significant difference in the preferred medium for children (*p* = 0.008) with 65% (*n* = 13) of parents reported their child preferred using the computer because the computer was the simplest and fun to use. The computer or paper assessments were perceived to be quicker than the handheld device by the majority of CYP (87%; *n* = 67; *p* = 0.001) and parents (91%; *n* = 21; *p* = 0.019). The majority of parents (91%; *n* = 21) also found the computer or paper to be easier to understand than the handheld device (*p* = 0.032) and 78% (*n* = 60) of CYP found the computer or paper more useful for describing pain than the hand-held device (*p* = 0.027). The majority of physicians (60%; *n* = 9) would recommend the computer-generated summary
Townsend (2020); USA; Non-randomised experimental study [68]	To present initial validity data on three web-based computerized versions of the Kiddie Schedule for Affective Disorders and Schizophrenia (KSADS-COMP)	*N* = *158*; Secondary Care; 9–17 years; Affective Disorders/ Schizophrenia	KSADS-COMP self-administered (child and parent)/KSADS-COMP clinician administered/ PHQ-9/ BCMS/GAD-7/SWAN/ Primary Care PTSD Screen; CYP and parent proxy-completion; half were randomly assigned to complete the adolescent and parent self-administered KSADS-COMP, and half were randomly assigned to complete the clinician-administered KSADS-COMP, those who met criteria for MDD, a bipolar diagnosis, ADHD, ODD or CD, PTSD, a substance use disorder, or no lifetime diagnoses during the first assessment were invited for a second study visit to complete the alternate version of the KSADS-COMP within three weeks	The following statements were rated as agree or strongly agree by CYP and parents respectively: I was comfortable answering questions on the computer (91%, 99%), The questions were clearly stated and understandable (85%, 94%), The computer did a good job asking me about my feelings (90%, 96%), I felt less embarrassed answering these questions on the computer than I would have with a clinician (71%, 54%), I found the computer interview to be a helpful process to go through (89%, 96%). Both CYP and parents expressed high satisfaction with the technical features of the self-administered KSADS-COMP, and of the user-friendliness of the technology. 85% of CYP stated they were willing to be interviewed by computer again when asked if they would prefer to be asked these types of questions by computer or clinician after completing the self-administered KSADS-COMP, 54% said computer, 11% said clinician, and 35% had no preference. Among the parents, 99% (*n* = 132) said they would be willing to be interviewed again by computer. In terms of interview preference, 28% of the parents stated they preferred the computer, 22% stated they preferred a clinician, and 50% had no preference
Uzark (2013); USA; Non-randomised experimental study [69]	To evaluate the clinical utility of health related QOL assessment in a paediatric cardiology outpatient clinic	*N* = *179*; Tertiary Hospital; 9–18 years; Cardiology	PedsQL 4.0 Generic Core Scales; CYP completion only; completed at a routine scheduled cardiology follow-up visit while waiting to see the cardiologist	Professionals felt information from QoL assessment was important and had a high impact on their practice including identifying concerns outside if physical functioning and influencing patient management decisions. The PedsQL was easy to use and understand and did not interfere with routine practice
van Bragt (2016); The Netherlands; Randomised Control Trial [88]	To assess the content of an intervention which integrates individual goals in outpatient clinic asthma management (based on self-management principles) of children 6–12 years of age	*N* = *42*; Tertiary Hospital; 6–12 years; Asthma	Pelican; CYP completion only; CYP complete the questionnaire online before appointments with the asthma nurse	Nurses thought that children were often unable to distinguish between current and recent problems and often relied on old memories, especially when asthma-related problems had had a substantial emotional impact. Nurses also mentioned that some parents thought their child was unable to oversee his/her asthma-related problems and to provide reliable answers without parental help Discussion: Children evaluated the Pelican instrument as easy to complete and fun to do
van der Merwe (2019); South Africa; Mixed Methods [102]	To describe the clinical utility and perceived value of a CCW-administered mHealth screening programme for early detection of developmental delays in vulnerable populations. Clinical utilities were examined in terms of referral rate, test duration and early detection	*N* = *148*; Community; 1–38 months; HIV/AIDS/ developmental delay	PEDS/PEDS:DM; parent proxy completion only; administered by CCWs in caregivers preferred language electronically	CCWs [*n* = 10] reported the benefits of the mHealth tools included early referral, the positive impact on the community and the importance of developmental screening and surveillance. CCWs also reported increased knowledge regarding typical development and the importance of developmental surveillance. The perceived value of the screening programme was highlighted including aspects such as time-efficiency, convenience, practicality and overall enjoyable experience. 100% of community care works strongly agreed that the mhealth tool and screening had a positive impact on the community. 90% strongly agreed that the App instructions were clear; 100% agreed or strongly agreed that they had adequate training; 80% agreed or strongly agreed that it was both easy to administer and easy to administer in homes in the community; 100% agreed or strongly agreed that it was quick to administer, that caregivers understood the questions, and that it provided accurate results; and 90% agreed or strongly agreed that caregivers agreed with final results. No one responded with the disagree or strongly disagree options
van Muilekom (2021); The Netherlands; Mixed Methods (Questionnaire results not included as age range of CYP was 12–19 years) [89, 90]	To provide insight into patients’ and parents’ perspective on the use of the KLIK PROM portal in order to optimize its implementation in paediatric clinical practice	*N* = *25*; Tertiary Hospital; 1–18 years; Juvenile idiopathic arthritis/Cystic Fibrosis/Cancer/Gastrointestinal diseases/Home parenteral nutrition/ Haemophilia/other chronic conditions	KLIK PROM; CYP and parent-proxy completion; PROMs on the profile are completed by CYP/parents before appointments and then discussed with clinician during appointment	Parents generally rated KLIK as positive in helping to prepare for consultations and provided insight into patients’ functioning, improving conversation content and adding value and efficiency to consultations. They felt that the KLIK was able to detect problems at an early stage and support was then able to be provided in a timely manner. Parents also liked the website layout, the security of the KLIK website is and how their data remains anonymous. Some patients and parents rated the content of PROMs positively, as they covered all important topics and are clear, but others felt that the questions in the PROMs are difficult to understand, repetitive and not relevant for every patient. Some patients rated completion time as good, but others felt it was time-consuming; there was ambiguity in responses about ease of use of KLIK and how often clinicians discussed results
Weidler (2021); USA; Qualitative [70]	To identify facilitators and barriers to implementing standardized outcome measurement in cleft care	*N* = *32*; Tertiary Hospital; children unspecified; Cleft Lip and Palate	N/A	Providers and staff viewed standardized outcome measurement as the gold standard and believed that care should be delivered according to those set standards. Providers also viewed standardized outcome measurement as being the preferred method/approach to collecting the clinical information needed to guide treatment decisions. Participants viewed standardized outcome measurement as a complex intervention that would require overcoming numerous existing and potential barriers to be successfully implemented
Weiner (2016); UK; Non-randomised experimental study [81]	To explore the extent to which young people and their family members engaged with the screening of psychological well-being and whether young people and their family report concerns during their follow-up appointments	*N* = *21*; Tertiary Hospital; 9–18 years; cancer	SDQ/SMFQ/EuroQoL/DT/EQ-VAS; parent proxy and CYP completion; questionnaires were completed after their clinic appointment with an assistant psychologist	CYP and parents rated the Questionnaires as interesting, relevant, and helpful and most found the follow-up appointments beneficial
Westergren (2021); Norway; Mixed Methods [109]	To examine how the core implementation components were adjusted for the “Starting Right” health service innovation, the success with tool adoption among staff in child and school health centres, and the success with tool acceptance among parents responding to health assessments	*N* = *208*; Primary Care; 2–6 years; healthy/development delay	SDQ/KIDSCREEN-27; parent-proxy report only; administered online	Professionals raised concerns around whether hard to reach families with the most need would respond to the questionnaires and the language barrier for those who would not be able to complete the measures in Norwegian. Professionals noted it was time consuming to register families on the online system, to distribute questionnaires and schedule appointments but the tool was considered useful to create a dialogue with families in appointments and parents were positive. There were concerns from professionals around data protection. Professionals reported that integration between the electronic patient record and the online tool would have enhanced more seamless and effective working processes and the systems not being joined up was a barrier to implementation
Windham (2014); USA; non-randomised experimental study [71]	To describe screening implementation challenges and results by demographic and instrument characteristics to aid in assessing effectiveness in the real-world setting of a low-income, Hispanic population and for interpreting surveillance findings	*N* = *1760*; Primary Care; 16–30 months; Autism	M-CHAT/ASQ; parent-proxy only; staff administered screens to parents in the waiting room	Professionals noted they did not have time to screen all their patients and that the ASQ was too long, so screens were often only used when Autism or other developmental concerns were already suspected. M-CHAT was endorsed by 85% of professionals for Autism screening as part of standard practice but that it was not as useful for Spanish speakers
Yamada (2017); Canada; Cross sectional [97]	To assess how organizational context moderates the effect of research use and pain outcomes in hospitalized children	*N* = *1743*; Tertiary Hospital; children unspecified; acute procedural pain	PIPP/FLACC/FPS-R/NRS; CYP and parent-proxy report; administered by professionals in hospital	Research evidence increased valid pain assessment use, as did strong leadership/implementation leads, and the organisational culture Pain assessment was greater in units with higher organizational context scores
Yu (2021); Canada; Quality Improvement [98]	to determine the equivalence of the paper and e-forms of CHAQ and QoML questionnaires and, identify potential benefits and barriers associated with using an e-form to capture PROMs, and gather feedback on user experience	*N* = *196*; Tertiary Hospital; paediatric unspecified; rheumatology	Caregivers CHAQ/ QoML; administered both on paper and electronically; parent proxy and patient completion	Barriers to completing the electronic versions of the measures included poor Wi-Fi connectivity and the limited number of devices available, the need to disinfect devices after use and the potential theft of devices. The cost per patient for each paper measure was $1.23. The overall cost for the electronic versions was $500, which included the two electronic tablets used to administer the measures. Cost savings would be realized after 407 uses which would take approximately four weeks in the clinic

*Abbreviations*: *ADHD* Attention Deficit Hyperactivity Disorder, *ALHIV* Adolescents living with HIV, *ART* antiretroviral therapy, *ASD* autism spectrum disorder, *ASQ-SE* Ages and Stages Questionnaire Social-Emotional, *BCMS* Brief Child Mania Rating Scale, *BHS-ED* Behavioural Health Screening–Emergency Department, *CAF*: Common Assessment Framework, *CALI* Children’s Activity Limitation Interview, *CAMHS* child and adolescent mental health services, *CAT* computer-adaptive testing, *CBT* Cognitive Behavioural Therapy, *CCW* Community care workers, *CD* Conduct Disorder, *CDI* Children’s Depression Inventory, *C-GAS* Children’s Global Assessment Scale, *CHAQ* Childhood Health Assessment Questionnaire, *CHI-ESQ* Commission for Health Improvement Experience of Service Questionnaire, *CHOIR* Collaborative Health Outcomes Information Registry, *CHU9D* The Child Health Utility 9D, *CiC* Children in Care, *CIMI* Clinical Improvement through Measurement Initiative, *CL/P* cleft lip/palate, *CPOS* Children’s Palliative care Outcome Scale, *CWS* Child Wellness Screening, *CYP* Children and Young People; DISABKIDS: Quality Of Life in children and adolescents with DISABilities and their Families; DM-Y: Diabetes Attitudes Wishes and Needs Monitoring Individual Needs in Young People With Diabetes-Youth; DT: distress thermometer; ECSA: Early Childhood Screening Assessment; e-form: electronic-form; EHR: Electronic Health Record; ePAT: electronic Psychological Assessment Tool; EPDS: Edinburgh Postnatal Depression Scale; eProfile: electronic Profile; ePROM: electronic patient-reported outcome measure; ePRO: electronic patient-reported outcomes; EQ-5D-Y: European Quality of Life 5 Dimension Youth; EQ-VAS: European Quality of Life Visual Analogue Scale; EuroQoL: European Quality of Life; FAPD: Functional abdominal pain disorders; FDI: Functional Disability Inventory; FLACC: Face, Legs, Activity, Cry, Consolability scale; FOCUS: Focus on the Outcomes of Communication Under Six; FPS-R: Faces Pain Scale-Revised; FVQ_CYP: functional vision questionnaire; GAD-7: Generalised Anxiety Disorder Assessment; GMFCS: Gross Motor Function Classification System; GMFM: Gross Motor Function Measure; HCP: health care professional; HEADSS: Home, Education, Activities, Drugs, Sexuality, Suicide/Depression; HITS-Domestic Violence Questions: Hurt, Insult, Threaten, and Scream- Domestic Violence Questions; HIV/AIDS: human immunodeficiency virus/ acquired immune deficiency syndrome; HoNOSCA: Health of the Nation Outcome Scale for Children and Adolescents; HP: health professional; HRQoL: Health related quality-of-life; ICF: International Classification of Functioning Disability and Health; INGO: International non- governmental organisation; ITC: Infant–Toddler Checklist; KIDSCREEN: SCREENing for and Promotion of Health Related Quality of Life in Children an Adolescents; KLIK: Kwaliteit van Leven In Kaart (Dutch: Quality of life in daily clinical practice); KSADS-COMP: Kiddie Schedule for Affective Disorders and Schizophrenia; KT: Knowledge Translation; LAUNCH: Linking Actions for Unmet Needs for Children’s Health; MBC: measurement-based care; M-CHAT: The Modified Checklist for Autism in Toddlers; M-CHAT-R: The Modified Checklist for Autism in Toddlers Revised; M-CHAT-R/F: The Modified Checklist for Autism in Toddlers Revised with Follow-up; MDD: major depressive disorder; MH: mental health; mhealth: mobile health; MoH: Ministry of Health; MY-Q: Monitoring Individual Needs in Young People With Diabetes-Youth Questionnaire; NGO: non- governmental organisation; NRS: Numerical Rating Scale; ODD: oppositional defiant disorder; PAT: Psychosocial Assessment Tool; PATrev: Psychosocial Assessment Tool adapted for the Canadian context; PCC: primary care clinician; PCIS: Parent Caregiver Involvement Scale; PCOM: Person-Centred Outcome Measure; PCS-C: Pain Catastrophizing Scale-Children; PCS-P: Pain Catastrophizing Scale-Parent; PDSA: Plan Do Study Act; PEDI: Pediatric Evaluation of Disability Inventory; PEDS: Pediatric Evaluation of Developmental Status; PEDS:DM: Parents’ Evaluation of Developmental Status-Developmental Milestones; Peds-CHOIR: Pediatric-Collaborative Health Outcomes Information Registry; PedsQL: Pediatric Quality-of-Life; PHC: primary healthcare centre; PHQ-9: Patient Health Questionnaire-9; PHQ-A: Patient Health Questionnaire modified for Adolescents; PIPP: Premature Infant Pain Profile; POSI: Parent’s Observation of Social Interaction; PPIS: Pediatric Pain Interference Scale; PPP: Pediatric Pain Profile; PQ: Pain-QuILT (quality, intensity, location, tracker); PRO: patient-reported outcomes; PROMIS: Patient-Reported Outcomes Measurement Information System; PROM: Patient-reported outcome measure; PSC: Pediatric Symptom Checklist; PSL: Preschool Speech and Language; PTSD: post-traumatic stress disorder; QI: quality improvement; QLIC-ON: Quality of Life In Childhood Oncology; QoL: quality-of-life; QOLLTI-F: Quality of Life in Life-threatening Illness-Family Carer questionnaire; QoML: Quality of My Life; ROM: routine outcome measurement; SCARED: Screen for Child Anxiety Related Disorders; SDQ: strengths and difficulties questionnaire; SEGC: Greenspan Social and Emotional Growth Chart; SEIQoL: Schedule for the Evaluation of Individual Quality of Life; SLP: speech-language pathologist; SMFQ: Short Mood and Feelings Questionnaire; SRH: sexual reproductive health; SUPER-KIDZ: Standardized Universal Pain Evaluation for pediatric rheumatology providers; SWAN: Strengths and Weaknesses of ADHD Symptoms and Normal Behavior Scale; SWYC: Survey of Well Being of Young Children; TAPQOL: TNO-AZL (Netherlands Organisation for Applied Scientific Research Academic Medical Centre) Preschool children Quality of Life; TUDS: Timed Up and Down Stairs Test; TUG: Timed “Up & Go” Test; UPROMISE: Using PROMs to Improve Service Effectiveness; VAS: Visual Analogue Scale; VQoL_CYP: vision-related quality of life

#### Study characteristics

##### Country

Of the *n* = 69 articles retained, *n* = 30 were conducted in the USA [42–71], *n* = 10 in the UK [72–81], *n* = 9 in The Netherlands [18, 82–90], *n* = 8 in Canada [91–98], *n* = 2 in Sweden [99, 100] and South Africa [101, 102], and *n* = 1 from each of Australia [103], Austria [104], Belgium [105], Germany [106], Iceland [107], Malawi [108], Norway [109], and North America (countries unspecified for the latter) [110].

##### Study design

With respect to study design, *n* = 17 studies used mixed methods [58, 65, 72, 75, 79, 82, 86, 89, 90, 95, 99, 100, 102, 103, 106, 107, 109, 110]; *n* = 15 studies used qualitative methods [46, 52, 55, 57, 60, 67, 70, 76, 78, 80, 91, 93, 94, 105, 108]. *N* = 12 studies were non-randomised experimental studies [44, 54, 61, 68, 69, 71, 77, 81, 85, 92, 96, 101], and *n* = 12 were quality improvement projects [42, 43, 45, 47–49, 51, 53, 62–64, 98]. There were *n* = 5 studies used cross-sectional designs [50, 59, 73, 74, 97], *n* = 4 case reports [18, 56, 66, 83], *n* = 3 cohort studies [84, 87, 104], and *n* = 1 study was part of a randomised control trial [88].

##### Setting

The most reported setting in articles was tertiary care hospital settings (*n* = 35) [18, 44, 46, 50–52, 54, 56, 58–61, 63, 69, 70, 79, 81, 82, 84–91, 94–98, 101, 103, 104, 106, 110], and *n* = 2 were conducted in both tertiary hospital and community settings [76, 105]. Of the *n* = 11 articles reporting on studies conducted in secondary care settings, these included: *n* = 8 mental health care settings [47, 62, 68, 72–75, 80], *n* = 2 speech and language clinics [92, 93], and *n* = 1 physiotherapy clinic [66]. A further *n* = 2 articles reported on studies conducted across secondary mental health care settings and the community [65, 77]. There were *n* = 12 articles reporting on studies conducted in primary care settings [42, 43, 45, 49, 53, 57, 64, 67, 71, 99, 100, 107, 109], and *n* = 2 articles reported on studies conducted across primary care and community settings [48, 55]. *N* = 2 articles reported on studies took place solely in the community [78, 108] and *n* = 1 took place across multiple settings, but did not specify details [83].

##### Population studied

Participants in included studies had a range of medical conditions, with many studies including children with multiple conditions. This included (using ICD-10 headings [111]): mental and behavioural conditions [42, 43, 45, 47–49, 54–58, 62, 64, 65, 67, 68, 71–75, 77, 78, 80, 92, 93, 99, 100, 102, 107, 109], cancer [18, 50, 59–61, 81, 83, 86, 87, 89, 90, 96, 103, 104], rheumatological conditions [50, 83, 84, 89, 90, 98, 106, 110], pain (chronic and acute) [44, 53, 63, 94, 95, 97, 110], endocrine conditions [46, 50, 51, 56, 82, 106], haematological conditions [50, 58, 61, 89, 90, 103], circulatory conditions (cardiac and pulmonological) [50, 56, 61, 69, 83], gastrointestinal conditions [50, 53, 89, 90], infectious diseases [83, 102, 108], respiratory conditions [56, 88, 106], neurological conditions [50, 95, 101], metabolic conditions [83, 89, 90], nephralgic conditions [56, 83], allergy/immunological conditions [56, 58], organ/stem-cell transplant [61, 91], other life-limiting/life-threatening conditions [76, 105], congenital conditions [70], ophthalmological conditions [79], and 4 studies included other chronic/unspecified conditions [52, 66, 85, 89, 90].

##### Measures studied and methods of completion

A range of generic and disease specific measures were used, including the Pediatric Quality-of-Life 4.0 (PedsQL 4.0), Psychological Assessment Tool 2.0 (PAT 2.0), and Quality Of Life in children and adolescents with DISABilities and their Families (DISABKIDS). There were *n* = 29 articles that reported on studies that included both child self-report and parent/caregiver-proxy report [18, 44, 47, 52, 56–58, 60, 61, 65, 66, 68, 72, 73, 81–86, 89–91, 95–98, 101, 105, 110]; *n* = 17 included child self-report only [45, 46, 51, 53, 54, 59, 62–64, 69, 79, 80, 88, 94, 104, 106, 108] and *n* = 18 included parent/caregiver-proxy report only [42, 43, 48, 49, 55, 67, 71, 77, 78, 87, 92, 93, 99, 100, 102, 103, 107, 109], usually because children were very young (< 6 years). There were *n* = 5 articles where this information was not reported [50, 70, 74–76].

### Study quality assessment

Included articles varied in quality. As CASP do not recommend or include a scoring system [30], articles assessed with the CASP checklists were unable to be scored, however, 90% [*n* = 45] of articles that were able to be scored were assessed as of good to high quality (Table 2)—i.e., those that could be scored met > 80% of criteria or had low-moderate risk of bias.

**Table 2:** Summary of quality appraisal scores

Study Design	Quality Appraisal Tool	Study	Score
Mixed Methods	Mixed Methods Appraisal Tool (MMAT) [32]	Westergren (2021) [109]	94%
		Barthel (2016) [106]	82%
		Batty (2013) [72]	100%
		Eilander (2016) [82]	100%
		Fäldt (2019) [99]	100%
		Fält (2020) [100]	100%
		Fullerton (2018) [75]	94%
		Herbert (2019) [58]	71%
		Jonsdottir (2020) [107]	88%
		McCarthy (2016) [103]	100%
		Orava (2019) [95]	77%
		Purbeck (2020) [65]	94%
		Robertson (2020) [79]	94%
		Schepers (2017b) [86]	100%
		Stinson (2012) [110]	76%
		van der Merwe (2019) [102]	94%
		van Muilekom (2021) [89, 90]	100%
Qualitative	Critical Appraisal Skills Programme (CASP) Qualitative Study Checklist [30]	Anthony (2021) [91]	N/A
		Brodar (2021) [46]	N/A
		Cox (2021) [52]	N/A
		Friedel (2020) [105]	N/A
		Godoy (2021) [57]	N/A
		Harding (2019) [76]	N/A
		Kazak (2017) [60]	N/A
		Kendall (2019) [78]	N/A
		Kip (2022) [108]	N/A
		Kwok (2022) [93]	N/A
		Lalloo (2014) [94]	N/A
		Sharples (2017) [80]	N/A
		Silver (2017) [67]	N/A
		Weidler (2021) [70]	N/A
		Fenikilé (2015) [55]	N/A
Non-Randomised Experimental	Risk Of Bias In Non-randomized Studies – of Interventions (ROBINS-I) [34]	Bhandari (2016) [44]	Low-Moderate
		Cunningham (2020) [92]	Low-Moderate
		Davies (2021) [101]	Low-Moderate
		Fein (2010) [54]	Low-Moderate
		Hardy (2015) [77]	Low-Moderate
		Kazak (2019) [61]	Low-Moderate
		Santana (2015) [85]	Low-Moderate
		Schulte (2019) [96]	Low-Moderate
		Townsend (2020) [68]	Low-Moderate
		Uzark (2013) [69]	Low-Moderate
		Weiner (2016) [81]	Low-Moderate
		Windham (2014) [71]	Low-Moderate
Quality Improvement Projects	Quality Improvement Minimum Quality Criteria Set (QI-MQCS) [33]	Berger-Jenkins (2019) [42]	100%
		Berry (2014) [43]	81%
		Bose (2021) [45]	100%
		Butz (2017) [47]	94%
		Campbell (2017) [49]	100%
		Campbell (2021) [48]	88%
		Corathers (2013) [51]	100%
		Cunningham (2018) [53]	94%
		Krishna (2019) [62]	94%
		Lynch-Jordan (2010) [63]	94%
		Mansour (2020) [64]	94%
		Yu (2021) [98]	81%
Cross-Sectional	Joanna Briggs Institute (JBI) Checklist for Analytical Cross Sectional Studies [31]	Bear (2021) [73]	100%
		Chen (2022) [50]	100%
		Edbrooke-Childs (2017) [74]	100%
		Hinds (2013) [59]	100%
		Yamada (2017) [97]	88%
Case Reports	Joanna Briggs Institute (JBI) Checklist for Case Reports [31]	Engelen (2010) [18]	57%
		Gerhardt (2018) [56]	100%
		Haverman (2014) [83]	43%
		Schreiber (2015) [66]	100%
Cohort Studies	Critical Appraisal Skills Programme (CASP) Cohort Study Checklist [30]	Haverman (2013) [84]	N/A
		Meryk (2021) [104]	N/A
		Schepers (2017a) (87)	N/A
Randomised Control Trial (RCT)	Critical Appraisal Skills Programme (CASP) RCT Study Checklist [30]	van Bragt (2016) [88]	N/A

All qualitative studies (*n* = 15) reported clear aims and methodology addressing the research question. Weaknesses related to failures to discuss the relationship between researchers and participants, and lack of details of ethical considerations and recruitment strategies. The *n* = 1 randomised control trial reported a clear aim, but some methodological decisions were lacking, and participant demographics were not reported. The *n* = 3 cohort studies had clear, focused aims, and well detailed methods, but lacked detail about confounding variables and attrition.

Mixed methods studies (*n* = 17) met between 70–100% of the MMAT criteria indicating they were generally of high methodological quality. Methodologically weaker studies lacked detail of the sample and risk of non-response bias. Cross sectional studies (*n* = 5) were well-reported and on average met 98% of the JBI criteria. Case reports (*n* = 4) met on average 75% of the relevant JBI criteria. Non-randomised experimental studies of interventions (*n* = 12) assessed with the ROBINS-I tool were of low-moderate risk of bias; usually due to low adherence to the intervention, however these data were extracted as it pertained to the review aims. Quality improvement projects (*n* = 12) met on average 93% of the QI-MQCS criteria. Main areas of weakness were lack of reporting of patient-health related outcomes and data on the sustainability or scalability of the project.

### Adapted-CFIR constructs

Table 3 details the adapted-CFIR domains and constructs extracted from the literature. The five most common subconstructs included complexity [*n* = 37], knowledge and beliefs about the intervention [*n* = 37], relative advantage [*n* = 31], patient needs and resources [*n* = 25], and available resources [*n* = 24]. Findings within each adapted-CFIR construct/sub-construct are presented below. Those constructs that were identified in less than three studies are not included in the narrative synthesis due to insufficient data [25]. Illustrative quotes are provided in Table 4 (reported as Q1, Q2, etc.).

**Table 3:** Factors identified using the Adapted-CFIR influencing PCOM implementation. Adapted from Damschroder [36] and Safaeinili [37]

Adapted CFIR domains and constructs	Description	Total, n (%)
I Intervention characteristics	Aspects of PCOMs that might affect implementation success in paediatric healthcare	
A. Intervention source [18, 83, 87, 99]	Stakeholders’ perception of the source of the PCOM—whether it has been developed internally or externally	4 (5.8)
B. Evidence strength and quality [50, 55, 70, 73, 92, 97]	Stakeholders’ perception of the strength of the evidence supporting the belief that the use of a new PCOM will have the desired outcomes e.g., improve care	6 (8.7)
C. Relative advantage [44–46, 48–50, 53–56, 61, 62, 64, 65, 69, 70, 72, 79, 80, 82, 83, 85, 89–91, 93, 100, 103, 105, 106, 108, 110]	Whether stakeholders perceive a new PCOMS as advantageous over current practice	31 (44.9)
D. Adaptability [42, 52, 55, 57, 72, 78, 94, 100, 106, 108, 109]	The degree to which a new PCOM can be adapted to current systems and practices of the healthcare setting	11 (15.9)
E. Trialability	The ability to test the use of a new PCOM on a small scale in the hospital or hospice first, and to be able to revert back to previous practice is necessary	0 (0)
F. Complexity [43, 44, 49, 52, 56–63, 65, 67–72, 75, 76, 78, 80, 82, 86, 88–90, 94, 99, 100, 102–104, 106, 108, 110]	Perceived difficulty or challenges for implementing and using a new PCOM in routine practice by stakeholders	37 (53.6)
G. Design quality and packaging [58, 60, 68, 98, 110]	Stakeholder perception of the presentation of the PCOM	5 (7.2)
H. Cost [43, 44, 62, 70, 98, 101, 108, 109]	Cost associated with implementing a new PCOM	8 (11.6)
II. Outer setting	External influences on implementation of a new PCOM	
A. Cosmopolitanism [43, 50, 55, 57, 76, 82, 106]	The degree to which the healthcare setting is networked with others	7 (10.1)
B. Peer pressure [70, 82, 106]	Pressure experienced by the healthcare setting to implement a new PCOM; typically, because most or other peer healthcare settings have already implemented	3 (4.3)
C. External policy and incentives [55, 80, 82, 92, 108]	External influences such as policy, regulations, recommendations, or public/benchmark reporting that encourage or discourage implementing a new PCOM in the healthcare setting	5 (7.2)
III. Inner setting	Characteristics of the implementing healthcare setting	
A. Structural characteristics [43, 50, 57, 75]	The social architecture, age, maturity, and size of the healthcare setting	4 (5.8)
B. Networks and communications [46, 61, 66, 91, 103, 107]	The nature and quality of social networks and communication within a healthcare setting	6 (8.7)
C. Culture [86, 97]	Norms, values, and basic assumptions of the healthcare setting	2 (2.9)
D. Implementation climate	The capacity for change and shared receptivity of individuals involved in implementing a new PCOM and the extent to which it will be rewarded, supported, and expected within the healthcare setting	
1. Tension for change [47, 55, 108]	The degree to which stakeholders perceive the need to change current practices	3 (4.3)
2. Compatibility [49, 55, 60, 61, 63, 69, 70, 82, 87, 93, 94, 98–100, 108]	The degree of fit between meaning and values attached to the use of the PCOM by healthcare professionals and families, and how those align with their own norms, values, perceived risks and needs, and the extent to which the introduction of a new PCOM fits with existing workflows and systems	15 (21.7)
3. Relative priority [47, 60, 91–93]	Individuals’ shared perception of the importance of implementation of a new PCOM within the healthcare setting	5 (7.2)
4. Organizational incentives and rewards [50, 55, 61, 67, 82, 108]	Incentives to increase and encourage the use of the PCOM in routine practice such as awards, recognition, or promotions	6 (8.7)
5. Goals and feedback [86]	The degree to which the goals of using a new PCOM are clearly communicated, acted upon, and fed back to staff, and the alignment of that feedback with the goals	1 (1.4)
6. Learning climate [75]	A climate in which: a) leaders express their own fallibility and need for team members’ assistance and input; b) team members feel that they are essential, valued, and knowledgeable partners in the change process; c) individuals feel psychologically safe to try new methods; and d) there is sufficient time and space for reflective thinking and evaluation	1 (1.4)
E. Readiness for implementation	Indicators of healthcare settings' commitment to the decision to implement a new PCOM into routine practice	
1. Leadership engagement [47, 57, 73, 86, 107, 108]	Commitment, involvement, and accountability of leaders and managers of the healthcare setting to the implementation	6 (8.7)
2. Available resources [42, 43, 46, 47, 53, 55, 57, 58, 60, 63, 65, 67, 71, 72, 80, 82, 86, 93–95, 98, 101, 108, 109]	The level of resources the healthcare setting dedicates to implementation and on-going use of the PCOM, including money, training, education, and time	24 (34.8)
3. Access to knowledge and information [18, 42, 46–48, 50, 52, 57, 64, 72, 80, 85, 92, 93, 95, 100, 108]	Ease of access to sufficient and appropriate information and knowledge about the PCOM and how to incorporate it into routine practice	17 (24.6)
IV. Individual characteristics	Individual beliefs, knowledge, and attitudes of stakeholders toward a new PCOM and its implementation	
A. Knowledge and beliefs about the intervention [44, 46, 49–51, 53, 55, 57, 58, 61, 67–74, 76, 78–81, 84–86, 89–92, 94, 100, 102–104, 106–108]	Individuals’ attitudes toward and value placed on PCOMs and familiarity with their use and impact on care	37 (53.6)
B. Self-efficacy [46, 50, 57, 73, 74, 79, 82, 93, 99, 108]	Healthcare professionals’ belief in their own capabilities to execute the course of action to achieve implementation goals, and children and parents’ belief in their ability to use the PCOM	10 (14.5)
C. Individual stage of change	The phase an individual is in as they progress toward skilled, enthusiastic, and sustained use of the new PCOM	0 (0)
D. Individual identification with organization [60, 78, 86]	Stakeholders’ perception of their relationship with the healthcare setting	3 (4.3)
E. Other personal attributes [42, 43, 50, 55, 57, 59, 60, 63, 67, 70, 72–74, 76, 83, 85, 87, 89–91, 93, 96, 99, 108]	Individuals’ attributes that affect implementation such as, motivation, values, experience, capacity, and learning style	14 (20.3)
V. Process	Stages of the implementation process that can impact its success	
A. Planning [43, 46, 56, 57, 60, 63, 64, 70, 78, 86, 93]	The degree to which guidance and tasks for healthcare professionals implementing a new PCOM intervention are developed and agreed upon in advance	11 (15.9)
B. Engaging	Attracting and involving stakeholders in the implementation of the PCOM through social marketing, education, training, or other similar activities	
1. Opinion leaders [60, 65]	Individuals in the healthcare setting who have a formal or informal influence on the attitudes and beliefs of others in relation to the implementation of a new PCOM	2 (2.9)
2. Formally appointed internal implementation leaders [43, 56, 57, 65, 97, 107]	Individuals from within the healthcare setting who have been formally appointed with responsibility for implementing the PCOM	6 (8.7)
3. Champions [57, 60, 62, 65]	Individuals who dedicate themselves to driving the implementation of the PCOM and overcoming indifference or resistance to using the PCOM in the healthcare setting	4 (5.8)
4. External change agents [65, 86, 107, 108]	Individuals from outside the healthcare setting who formally influence or facilitate the implementation of the PCOM in a desirable direction e.g., commissioners of healthcare services	4 (5.8)
C. Executing [42, 52, 61, 63, 64, 86, 100]	Carrying out or accomplishing the implementation of the PCOM into routine practice according to plan	7 (10.1)
D. Reflecting and evaluating	Quantitative and qualitative feedback about the progress and quality of the implementation of the PCOM accompanied with regular personal and team debriefing about progress and the experience of using the PCOM	0 (0)
VI. Patient needs and resources [45, 46, 48, 53, 54, 56, 57, 60, 63, 64, 69, 70, 76, 77, 82, 87, 89–92, 100, 102, 103, 105, 106, 108]	The extent to which children’s needs, as well as barriers and facilitators to meet those needs, are known and prioritized by the healthcare setting	25 (36.2)

**Table 4:** Illustrative quotes

Quote Number	Quote
Intervention Characteristics	
Q1	“we do not want to commit to anything extra than just the standard treatment” – Parent of Child with Cancer [87]
Q2	‘It would be really nice if there was more pediatric evidence that one could actually rely on’ – Family physician [55]
Q3	“it would provide us with information that we may not be getting. Especially when the dynamics – when there’s a parent in the room and there’s a child – you might be getting… what’s important to the parent as opposed to what’s important to the child. [PROMs] may help highlight them to us” – Healthcare practitioner [91]
Q4	“Very few of us use it [the FOCUS], at all really. It doesn’t drive our therapies it doesn’t drive our strategies we will recommend, it doesn’t really drive anything we do at this point so, so then is really is, it becomes administrative’ – Speech-Language Pathologist [93]
Q5	“The other thing that was very helpful is getting our institution to actually provide institutional support specifically getting the Smart Form built into our EMR. I thought that was critical…”—Pediatric primary care clinician [57]
Q6	“the tool [developed and validated in the USA and implemented in Malawi without further validation] should include questions that are specifically for Malawi that are culture specific”—Psychosocial Counsellor Mentor [108]
Q7	“I found it easy to use and really helpful” – Adolescent with Chronic Pain [94]
Q8	“From the parents’ side is it’s too long. And unfortunately, the reading skills of our families is not, you know, up to speed to what’s there [...]” – Pediatric Attending [67]
Q9	“they tore out the pages or crossed it [items of a sensitive nature] out and just threw it [the PCOM] away” – Child Health Centre Nurse [100]
Q10	“My daughter usually says ‘Oh no, not again’ when she is handed the paper format. She loved using the tablet format. It is much more user friendly for kids/ teens.” – Parent/Caregiver of child with rheumatological condition [98]
Q11	“I think that... it will just be energy spent trying to get measures rather than trying to connect with the patient.” – Plastic surgeon [70]
Outer Setting	
Q12	‘If there are resources to refer them to, sure. But I don’t want to screen for something I can’t get services for.’ – Family Physician [55]
Q13	“it should not be introduced by an NGO because if it is being introduced by an NGO the health providers will consider it as an NGO thing. So, this should be incorporated into the main system of the government. The clinicians at government level need to incorporate this” – Medical Technician Mentor [108]
Inner Setting	
Q14	“I think it is important that everybody’s sharing the same tool.” – Oncologist [103]
Q15	“I think it is really helpful just to see a bit of background information about them [the families] without necessarily having to go in and find that we’re all asking the same questions” – Child Life Therapist [103]
Q16	“Well, I think the downside is maybe just the burden and the work involved in making everything very systematic” – Orthodontist [70]
Q17	“... the parents fill it in themselves … I just kind of leave it with the families. I just take a few minutes to explain that, you know, it’s just three pages, it’s just a way for me to get to know you and your family. So for me it’s pretty minimal and even the scoring doesn’t take all that long really.” – Health and Social Care Professional [60]
Q18	“I think once healthcare team members see … that there are results that we can use out of this, I think certainly our practitioners would be very interested in this.” – Health and Social Care Professional [60]
Q19	“Implementation of new things usually is accommodated when there is an incentive attached to it, from experience that’s what I have seen” – Adolescent District Coordinator Mentor [108]
Q20	‘…we don’t get paid enough and that’s what it comes down to, reimbursement. You should get decent reimbursement for the time that it takes …’ – primary care physician [55]
Q21	“As management we do support because when any new program comes, there is a management meeting, and we call general staff meeting explaining that we have this program…… Since management here receives programs well and it gives the program well to the service providers, the service providers also takes the initiative well and implement it.” – Deputy Clinic Manager Mentor [108]
Q22	“I don’t think that we’re appropriately staffed for successful child wellness screening…I think it should be people who have—are a little bit more consistent in work schedules so that they come to work reliably. Um, and that they have better organizational skills and better communication skills.” – Implementation Lead [67]
Q23	“Space is inadequate. The health providers would want to do some activities but where would they sit? The issues of HIV are sensitive, and you can’t just sit anywhere?” – Health Surveillance Mentor [108]
Q24	“It’s still a challenge to figure out how to gather the data and enter it live with the client.” – Child Mental Health Professional [65]
Q25	“We do put a reminder in our booking. So the therapist does have to remember that [to input the reminder into the booking system]. But in the booking, we set a reminder to booking. It’s noted and it comes up on the day’s log sheet. So when they walk into a session, it says the time the date, and the FOCUS. And so she [the SLP] knows they [the family] need to have the FOCUS done.” Speech-Language Pathologist [93]
Individual Characteristics	
Q26	“unexpected responses [to the PCOM not elicited through standard history taking] significantly influenced patient management decisions” – Cardiologist [69]
Q27	“[If asked to complete PROMs] I’d feel annoyed… I feel it’s easier to talk about the things” – Child receiving solid organ transplant [91]
Q28	“I did worry, cos I thought … when I read through the questions, I thought he had to do it, all of them, and I thought, ‘Oh, my God, he's really slow.’” – Parent [78]
Q29	“With this you are working together with parents … you are encouraging the parents to have their own assessment with their child and see where they are before they come and see you.” – Health Professional [78]
Q30	“I think it is not always necessary to complete the KLIK questionnaires” – Child [89, 90]
Q31	“The clinician often does not discuss the KLIK ePROfile” – Parent [89, 90]
Q32	“too many other things on our mind [to complete the PCOM” – Parent of Child with Cancer [87]
Q33	“I mean, my child’s an introvert. So, I think sometimes [disclosing] is really uncomfortable for [them]” – Caregiver of Child with Solid Organ Transplant [91]
Process	
Q34	“There are multiple steps where the screening process can sometimes not go well and so then we have to go back and fix it. So, we’ll go in and they wouldn’t have gotten the screen, so we have to get the screen. And then they wouldn’t have given the MA [Medical Assistant] the screen to score and we have to do that. And then, that’s just going outside the room after the visit has started and finding the appropriate person, and so that can be really inefficient.” – Pediatric Primary Care Clinician [57]
Q35	“this to me was sold to me ‘this is what you do now’” – Health Professional [78]
Q36	“First thing that we did was myself and the medical director, we went to the nursing leadership and we went to the administrative leadership in the clinic…we're all one team and we all kind of have our own tiers of leadership and we said, ‘You know, we are going to be doing this project. Do we get buy-in from you all to move forward with this project?’…that was kind of like the jumping point for going into the QI project.” – Pediatric Primary Care Clinician [57]
Q37	“I feel like we had a strong internal champion who kept us on task in a way that was not intrusive, but very supportive.” – Clinician [65]
Q38	“I think the biggest thing that helped was actually having a multidisciplinary team sort of as the champion for it in the clinic…by having one of your peers be a represented champion, that sort of got the other peers.” – Pediatric Primary Care Clinician [57]
Q39	“[External Change Agent] was very good about sharing resources that maybe she and one of the other sites came up with and having that accessible to other sites to use as kind of a framework to start with.” – Clinician [65]
Q40	“Parents think there is a lot to fill in and have often forgotten to fill it in and bring it with them” – Nurse [100]
Patient Needs and Resources	
Q41	“[A patient] would feel like their voice is being heard and they’re actually having a chance to say what they think and what they feel because you don’t always have a chance to do that in clinic” – Healthcare Professional [91]

#### Intervention characteristics

##### Intervention source

Professional engagement in the development process of the measure [18] and the perceived security of the platform hosting electronic- or e-PCOMs were both factors that facilitated implementation [83]. However, low rates of parent completion were observed when newly implemented PCOMs were introduced to participants in the context of a research study rather than as new aspect of routine clinical care [Q1] [87, 99].

##### Evidence strength and quality

Presenting evidence to support PCOM use and perceptions of PCOMs as the ‘gold standard’ were key facilitating factors for implementation and frequent continuous use; training/education programmes that emphasised that PCOMs were research-evidenced, valid, and reliable supported this [70, 73, 92, 97]. Similarly, a significant barrier to implementation was professionals’ perceptions that there was insufficient evidence justifying PCOM use or supporting them as valid instruments [Q2] [50, 55].

##### Relative advantage

The use of PCOMs was perceived as advantageous, particularly from professionals’ perspectives. One study reported that 80% [*n* = 53] of parents found PCOM use provided added value over standard consultations [82]. Advantages included: improving communication, engagement, and decision-making with patients and families [Q3] [53, 55, 56, 61, 69, 79, 80, 91, 100, 103, 106, 108], enhancing quality of care and assessment [44, 46, 53, 56, 61, 62, 69, 70, 79, 80, 82, 85, 100, 105, 108], identifying concerns that would have remained unidentified in standard consultations [45, 46, 49, 53, 54, 56, 64, 79, 82, 100, 103, 105, 106], and increased referral rates and access to other services and treatment [45, 46, 48, 53, 56, 64, 103].

Three studies reported that professionals continued to use PCOMs after studies ended due to improved identification of patients’ unmet needs and due to the PCOMs having become integrated into routine practice [49, 64, 100]. Where professionals did not consider PCOMs beneficial, this was often due to them being perceived as bureaucratic exercises that did not elicit new information compared to standard consultations [Q4] [50, 55, 72, 93, 103].

Regarding e-PCOMs, there were mixed perspectives as to whether professionals felt that technology enhanced workflow and assessment compared to traditional paper-based PCOMs [49, 65]. However, there was a strong preference for accessing reports and scores from measures electronically [49, 50, 89, 90, 110], while inclusion of visual representations of progress, e.g. graphs tracking scores over time, was considered beneficial [44, 83].

##### Adaptability

Where PCOMs could be integrated into electronic systems or platforms this facilitated implementation [Q5] [57, 94, 106]. Correspondingly, this was identified as a barrier in studies where integration did not occur [42, 55, 72, 109]. Lack of cross-cultural validity of PCOMs and those not provided in service users’ language were identified as significant barriers [Q6] [52, 57, 78, 100, 108, 109].

##### Complexity

Ease of PCOM use facilitated implementation, which included professionals’ views of administering the measure, interpreting the score, and feeding back scores to patients and families [52, 57, 61–63, 65, 69, 71, 75, 78, 80, 82, 94, 100, 102, 106, 108, 110]. The importance of measures being child/user friendly was emphasised including ease of completion [Q7] [44, 49, 52, 58, 59, 61, 68, 70, 76, 78, 80, 86, 88–90, 94, 99, 100, 103, 104, 106, 108, 110], appropriate measure length [56, 58, 65], and language and a reading level understandable to children and parents completing the measures [Q8] [43, 59, 60, 67, 72, 76, 99]. However, the content of PCOMs (particularly items of a sensitive nature) was a barrier to implementation [Q9] [65, 80, 100].

##### Design quality and packaging

There was general preference for digital administration methods, such as tablets or computers [Q10] [58, 60, 98, 110]. Yu et al. [98] found 83% [*n* = 196] of parent/caregivers preferred the electronic version over paper or had no preference. Similarly, Stinson et al. [110] reported that only 16% [*n* = 77] of children preferred pen and paper as the method of administration. Children preferred using technology to complete measures (compared to professional administration), with 71% [*n* = 112] responding that they agreed or strongly agreed with the statement ‘I felt less embarrassed answering these questions on the computer than I would have with a clinician’ [68].

##### Cost

The two most significant costs associated with implementing PCOMs were monetary cost and time. Costs of implementing and maintaining e-PCOMs were discussed [43, 44, 98, 101, 108], although the initial cost of e-PCOMs could be offset over time (due to the recurring costs of paper-based measures [98]). There were some reports that measures were time-consuming to administer during appointments and concerns that this might detract from dedicated patient care [Q11] [43, 70, 108, 109]. However, in further study, providers reported that once PCOMs were implemented into routine care, an average of 16 min of time was saved per appointment [62]. This was particularly important as those time-savings were able to be redirected to improving patient care [62].

#### Outer setting

##### Cosmopolitanism

Multi-disciplinary, joined-up, inter-agency working was a significant factor in implementation, as there are often many agencies and services involved in the care of children [76]. Partnerships between settings facilitated the implementation and sustained use of measures and this was linked to peer pressure sub-construct [82, 106]. A lack of resource to address identified unmet need was a significant barrier to sustained use [Q12] [43, 50, 55, 57].

##### Peer pressure

Linking to cosmopolitanism and partnerships between settings, if other clinics they worked with were using specific PCOMs, this increased the motivation of professionals to also use them [70, 82, 106]; one study reported this was a motivating factor for 86.1% [*n* = 31] of paediatricians [82].

##### External policy and incentives

External recommendations, guidelines, or association endorsements were a motivating factor for settings to implement and use PCOMs in practice [80, 82, 92]. However, the source of the recommendation could potentially impact implementation [Q13] [108]. Lack of awareness of or disagreement with recommendations from professional associations was a barrier [55].

#### Inner setting

##### Structural characteristics

The main barriers regarding structural characteristics of organisations were related to organisational changes such as high staff turnover [43, 57, 75]. Age of professionals also impacted perceptions of using PCOMs in routine practice; in one study, older practitioners were more likely to be sceptical about the validity and evidence-base for using PCOMs [50].

##### Networks and communication

Multidisciplinary team communication was seen as a prerequisite to support use of PCOMs [91, 107]. Professionals recognised that using PCOMs supported information sharing between staff in a more systematic way, which improved care [Q14, Q15] [46, 61, 66, 103].

##### Tension for change

One potential barrier to the implementation of PCOMs was staff readiness and willingness to change current practice [55, 108]. However providing education and training to staff on the expected benefits of using PCOMs could change attitudes and willingness to change and thus facilitate implementation [47].

##### Compatibility

Perceived disruption to workflows was a potential barrier to implementation [Q16] [55, 60, 70, 93, 94, 98–100]. However, in practice, the introduction of PCOMs was generally not seen as disruptive and they became an integral aspect of routine care [Q17] [49, 60, 61, 63, 69, 82, 87, 108].

##### Relative priority

Shared recognition of the importance of using PCOMs, sometimes referred to as ‘buy-in’ or ‘ownership’ [60], was considered an important facilitating factor for implementation and use of PCOMs in practice [Q18] [60, 93]. Where professionals or patients did not perceive the benefit of PCOMs, this was a barrier to implementation [47, 91]. Education and training may have the potential to facilitate implementation where perceptions of PCOMs are a barrier as one educational intervention increased speech-language pathologists’ positive perceptions of outcome measurement from 49% of participants to 71% of participants [*n* = 46] [92].

##### Organisational incentives and rewards

Monetary incentives or rewards were a potential motivating factor for professionals to use PCOMs in practice, particularly in lower-middle income [112] countries [Q19] [82, 108]. Lack of reimbursement for administering tools was a barrier to implementation and continued use, particularly in countries with privatised insurance-based healthcare [Q20] [50, 55, 61, 67].

##### Leadership engagement

Commitment and support from leadership significantly contributed to the successful implementation of PCOMs into routine practice. High levels of support from leadership was more likely to enable successful implementation than when they had different priorities [Q21] [47, 57, 86, 107, 108], although this was not always the case [73].

##### Available resources

Lack of resources was a major barrier to implementation [43, 53, 55, 67, 72, 93] and continued funding was a necessity for sustainability [67, 108]. Lack of time to implement, administer, score, and record results of measures was a significant barrier, and often remained a barrier even when other barriers had been addressed [46, 53, 55, 57, 60, 63, 71, 80, 86, 93, 95, 109]. Inadequate staff numbers and high staff turnover was a barrier[Q22] [47, 57, 67], while recruiting additional staff to support measure implementation and use was a facilitator [46, 47, 57, 80, 93]. Challenges finding physical spaces for patients and families to complete measures in private was also an issue [Q23] [58, 60, 67, 82, 93, 108].

The technology requirements of e-PCOMs often created challenges, specifically in relation to internet access, access to and cost of devices [65, 67, 80, 94, 98, 101]. Conversely, paper-based measures had posed challenges pertaining to PCOM availability, stationery resources [42, 93, 94, 108], and the additional time required to enter results into electronic patient records [Q24] [65].

##### Access to knowledge and information

Lack of awareness and knowledge of both PCOMs and how to incorporate them into routine practice was a barrier to implementation and sustained use [50, 57, 64, 72], as was more knowledgeable staff not sharing their knowledge with those less knowledgeable [108]. Successful strategies to address issues of knowledge about using PCOMs in routine practice included reminders of when and to whom to administer PCOMs, through electronic health records or emails [Q25] [42, 48, 64, 93, 95]. Ongoing efforts to engage professionals through additional training, webinars, handbooks, and guidelines, improved sustainability [46, 47, 52, 57, 64, 72, 80, 85, 92, 100]. Explanations for children and families completing measures regarding what PCOMs are, their purpose, and how to complete them also facilitated use of PCOMs in practice [18].

#### Individual characteristics

##### Knowledge and beliefs about the intervention

When PCOMs were perceived positively, for example as validated tools that could support assessment and improve care outcomes, this acted as a facilitating factor [Q26] [44, 46, 49, 51, 53, 57, 58, 61, 68–71, 73, 74, 78, 79, 81, 84–86, 89–92, 94, 100, 102–104, 106–108]. Conversely, when PCOMS were perceived more negatively by either professionals or children and families (for example as time consuming), this acted as a barrier [Q27] [46, 50, 55, 67, 72, 74, 80, 91, 100, 108]. Educational strategies were often key to supporting implementation and use [72, 80, 85, 92, 100]. Additionally, if parents of children felt PCOMs were being used as tests, this could create unnecessary stress for families and act as a barrier [Q28] [76, 78].

##### Self-efficacy

Professional confidence [57, 82, 108] or lack of confidence [50, 57, 79, 93] in using PCOMs was a respective facilitator or barrier. Training and education to use PCOMs could increase self-efficacy and support implementation [46, 74, 108]. As professionals gained experience using PCOMs in practice, their self-efficacy increased [73, 99].

##### Individual identification with organization

Challenging relationships between professionals and management and a perceived lack of organisational commitment to the intervention were a barrier reported by one study [86]. Trusting relationships between professionals and families, and opportunities to work in partnership facilitated implementation [Q29] [60, 78].

##### Other personal attributes

Several personal traits were identified that could influence successful implementation and routine use in practice. Following through on actions was an issue for parents in terms of remembering to complete and return screening forms [42], and for professionals in terms of administering measures and discussing results with patients [42, 63, 83, 85, 93]. Professionals’ confidence, experience, and discipline all had the potential to act as barriers or facilitators [50, 55, 57, 73, 74]. Motivation of professionals and families was also important [Q30, Q31] [85, 89, 90]. For parents and patients particularly, motivation was often linked to the perceived added value of the measure for the consultation. Other personal attributes that could impact implementation included parental mental load and stage of treatment/diagnosis (which were often linked) [Q32] [87, 96] and how comfortable children felt talking to professionals [Q33] [91].

#### Process

##### Planning

Clearly defined responsibilities that have been collaboratively agreed with advanced notice are important for successful implementation of PCOMs [43, 46, 56, 57, 60, 63, 64, 70]. Absence of planning presents potential barriers to adherence [Q34] [57, 64, 93]. The way in which PCOMs are introduced and formally ratified by managers is also likely to have an impact on the success and uptake [Q35] [78, 86].

##### Formally Appointed Implementation Leads

Formally appointed implementation leaders or teams and support of site leadership were seen as essential components to adoption and uptake of newly implemented outcome measures [Q36] [56, 57, 65, 97, 107]. Lack thereof was noted as a significant barrier [43]. This sub-construct had significant cross-over with the Champions sub-construct, as the terms were sometimes used interchangeably.

##### Champions

Individual or team champions were seen as playing a key role in raising awareness of the interventions and promoting the use and value of PCOMs and supporting colleagues [Q37, Q38] [57, 60, 62, 65].

##### External change agents

External change agents who provide support, in terms of policy, advice, resources, or other forms of support to assist implementation, were seen as a facilitating factor [Q39] [65, 86, 107, 108].

##### Executing

As noted in some of the previously discussed sub-constructs, there were a number of logistical, resource, and education/information barriers which resulted in the intervention not being used according to plan; addressing these barriers was found to reduce these issues and increase adherence [42, 61, 63, 64, 86, 100]. However, patients and families forgetting or being unable to complete and return measures or completing the wrong measure was also an issue impacting implementation of PCOMs [Q40] [42, 52, 63, 100].

#### Patient needs and resources

Patient needs were better identified with the introduction of PCOMs into routine practice. PCOMs identified concerns of children and families that professionals perceived would not have been picked up in standard practice [Q41] [45, 46, 53, 54, 57, 60, 63, 64, 69, 77, 82, 87, 89–92, 102, 103, 105, 106, 108], with one study noting a 68% increase in identification [54]. PCOMs also increased referral rates though identifying unmet needs [45, 48, 53, 64]. Improvements in HRQoL scores were attributed to PCOMs supporting treatment decisions in one study, which reported 33% improvement in scores [56]. Increased focus on children, and better provision of individualised person-centred care were also noted [70, 76, 100, 105].

### Evidence of effectiveness

Although several barriers to implementation were identified, numerous strategies from high quality research were able to successfully address barriers and support implementation of PCOMs into routine practice. In particular, training or educating professionals, children and families generally had a positive effect [42, 44, 47, 61, 63, 64, 73, 74, 80, 85, 92, 100] on the implementation of PCOMs, as did addressing logistical barriers [42, 63, 64]. Numerous studies also showed increased identification of concerns and referral rates after implementation of PCOMs [45, 46, 53, 54, 57, 60, 63, 64, 69, 77, 82, 87, 89, 90, 92, 102, 103, 105, 106, 108] which also acted as a facilitator for implementing PCOMs.

### Logic model for implementing person-centred outcome measures in paediatric healthcare settings

**Fig. 2:**
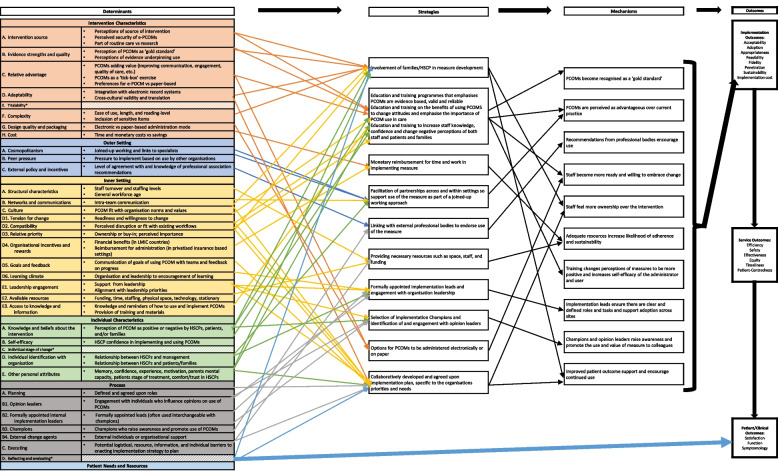
Logic model for implementing PCOMs into paediatric healthcare settings. Adapted from Smith et al. (2020) [38]

The findings of this review have informed the development of a logic model (Fig. 2) which identifies determinants, strategies, and mechanisms for implementation from these barriers and facilitators. The logic model illustrates how the existing evidence for determinants of implementation can be used to develop strategies to achieve implementation, service, and patient/clinical outcomes. It also demonstrates the mechanisms though which these interconnected factors achieve outcomes.

## Discussion

This review has identified key barriers and facilitators to the implementation of PCOMs into paediatric healthcare practice using the adapted-CFIR. These findings informed the development of a logic model that can inform and support future development of context-specific implementation strategies for implementing PCOMs in different paediatric settings.

Relative advantage of PCOMs were echoed in the adult evidence base [13, 16] and some systematic reviews of measures used in specific paediatric settings [11, 20, 22], demonstrating benefits to decision-making, communication, identification of concerns, patient quality of life and referrals.

Existing evidence on implementation, emphasising the importance of PCOMs being evidence based, valid, and reliable [13, 14, 22] is reflected in the sub-constructs of intervention source and avidence quality. Øvretveit et al. [14] note the importance of measures being developed with the adult patients using them, to ensure suitability. A systematic review by Coombes et al. [8] suggests, in line with the findings from this review, that children generally prefer computerised measures and highlights the importance of measures being developmentally appropriate (relating to language used, recall period, and response formats) [8]. This further evidences the importance of involving key stakeholders in the development of PCOMs to support implementation and the mechanisms through which this occurs can be seen visually in the logic model.

The access to knowledge domain was intrinsically linked to relative advantage, intervention source, and evidence quality. In order for the intervention source, evidence quality, and relative advantage to act as facilitators, professionals, patients, and families must be supported to understand the reliability, validity and benefits of the PCOM [14, 18, 22].

Findings relating to resources, staffing, and leadership are consistent with literature from adult healthcare services, including the importance of integration within existing systems and workflows, staff willingness to change and ‘buy-in’, and leadership engagement and support [13, 14, 18].

### Strengths and limitations

This systematic review provides a thorough, theory driven examination of the evidence for implementing PCOMs into paediatric healthcare settings. The adapted-CFIR supported the identification of facilitators and barriers to implementation, with only three sub-constructs for which there were no data identified. This supported the development of a comprehensive and theoreticallyinformed logic model. Given that 67% [*n* = 46] of the retained studies included child self-report measures, this review supports prioritisation of children’s voices in their care, and the centrality of person-centredness to quality care.

Of the sub-constructs for which no data was identified (trialability, individual stage of change, and reflecting and evaluating), it could be that these domains are not relevant to paediatric healthcare, or it could be due to limitations of the existing evidence base. These areas should be prioritised in future research.

### Recommendations for practice

From the evidence synthesis and logic model development, several strategies for implementing PCOMs into paediatric healthcare settings have been identified. Education about the benefit of PCOMs is important to increase professional’s understanding of the importance and benefit of PCOMs to facilitate implementation [72, 80, 85, 92, 100]. Including key stakeholders in measure development helps to ensure the outcomes being measured are relevant and useful [8, 14, 18]. This further precipitates a sense of shared ownership with professionals, patients and families over the PCOM being implemented [60, 93]. The identification of context-specific factors (such as financial incentives as a facilitator in lower-middle income countries or reimbursement in privatised insurance-based healthcare systems) [50, 55, 61, 67, 82, 108, 112] further demonstrates the importance of professionals’ understanding the context in which implementation occurs.

## Conclusions

To our knowledge, this is the first systematic review conducted into the implementation of PCOMs in paediatric healthcare that is not condition or setting specific. This review provides a comprehensive overview of the potential barriers to implementing and using PCOMs in paediatric healthcare, and the factors that can facilitate implementation and adherence.

This review has also demonstrated the suitability of the adapted-CFIR to theoretically inform implementation research in paediatric settings. The visual presentation of the logic model clearly demonstrates the interconnectedness of the numerous determinants of implementation. It also demonstrates the mechanisms through which implementation strategies can facilitate the implementation of PCOMs into paediatric healthcare settings to achieve improved outcomes for children and their families.

Using PCOMs in routine paediatric care is key to child-centred quality care. This review provides important evidence for how to implement PCOMs in practice in order to support better identification of patient needs. Future research should aim to assess the applicability and feasibility of this logic model in different settings to support implementation interventions, particularly in lower-middle income settings as much of the existing evidence come from higher income countries.

## Supplementary Information

**Additional file 1.**

**Additional file 2.**

**Additional file 3.**

**Additional file 4.**

## Data Availability

Not applicable.

## Abbreviations

PCOM: Person-Centred Outcome Measure
PRISMA: Preferred Reporting Items for Systematic Reviews and Meta-Analyses
PROSPERO: International Prospective Register of Systematic Reviews
NHS: National Health Service
CASP: Critical Appraisal Skills Programme
JBI: Joanna Briggs Institute
MMAT: Mixed-Methods Appraisal Tool
QI-MQCS: Quality Improvement Minimum Quality Criteria Set
ROBINS-I: Risk Of Bias In Non-randomised Studies—of Interventions
CFIR: Consolidated Framework for Implementation Research
HRQoL: Health Related Quality-of-Life
