# Supplementary material for: Implementation of child-centred outcome measures in routine paediatric healthcare practice: a systematic review
Source: Health Qual Life Outcomes. 2023 Jul 3;21:63. doi: 10.1186/s12955-023-02143-9 (PMC10316621; doi:10.1186/s12955-023-02143-9)
Supplement: Supplementary file 2 — Additional file 2. [file 12955_2023_2143_MOESM2_ESM.docx]

## S2: Embase Search

Embase <1974 to 2022 March 24>

1 Child*.mp. [mp=title, abstract, heading word, drug trade name, original title, device manufacturer, drug manufacturer, device trade name, keyword heading word, floating subheading word, candidate term word] 2935502

2 P?ediatric*.mp. [mp=title, abstract, heading word, drug trade name, original title, device manufacturer, drug manufacturer, device trade name, keyword heading word, floating subheading word, candidate term word] 726585

3 adolescen*.mp. [mp=title, abstract, heading word, drug trade name, original title, device manufacturer, drug manufacturer, device trade name, keyword heading word, floating subheading word, candidate term word] 1766776

4 teen*.mp. [mp=title, abstract, heading word, drug trade name, original title, device manufacturer, drug manufacturer, device trade name, keyword heading word, floating subheading word, candidate term word] 46108

5 young people.mp. [mp=title, abstract, heading word, drug trade name, original title, device manufacturer, drug manufacturer, device trade name, keyword heading word, floating subheading word, candidate term word] 44215

6 infant.mp. [mp=title, abstract, heading word, drug trade name, original title, device manufacturer, drug manufacturer, device trade name, keyword heading word, floating subheading word, candidate term word] 785751

7 exp Child/ 2859079

8 exp Pediatrics/ 117238

9 exp Adolescent/ 1656631

10 exp Infant/ 1063241

11 1 or 2 or 3 or 4 or 5 or 6 or 7 or 8 or 9 or 10 4443458

12 outcome measure*.mp. [mp=title, abstract, heading word, drug trade name, original title, device manufacturer, drug manufacturer, device trade name, keyword heading word, floating subheading word, candidate term word] 332139

13 symptom measure*.mp. [mp=title, abstract, heading word, drug trade name, original title, device manufacturer, drug manufacturer, device trade name, keyword heading word, floating subheading word, candidate term word] 1698

14 PRO.mp. [mp=title, abstract, heading word, drug trade name, original title, device manufacturer, drug manufacturer, device trade name, keyword heading word, floating subheading word, candidate term word] 366006

15 PCOM.mp. [mp=title, abstract, heading word, drug trade name, original title, device manufacturer, drug manufacturer, device trade name, keyword heading word, floating subheading word, candidate term word] 503

16 PROM.mp. [mp=title, abstract, heading word, drug trade name, original title, device manufacturer, drug manufacturer, device trade name, keyword heading word, floating subheading word, candidate term word] 6438

17 patient reported outcome*.mp. [mp=title, abstract, heading word, drug trade name, original title, device manufacturer, drug manufacturer, device trade name, keyword heading word, floating subheading word, candidate term word] 57833

18 patient cent?red outcome measure*.mp. [mp=title, abstract, heading word, drug trade name, original title, device manufacturer, drug manufacturer, device trade name, keyword heading word, floating subheading word, candidate term word] 300

19 (self adj2 measure).mp. [mp=title, abstract, heading word, drug trade name, original title, device manufacturer, drug manufacturer, device trade name, keyword heading word, floating subheading word, candidate term word] 8717

20 (proxy adj2 measure).mp. [mp=title, abstract, heading word, drug trade name, original title, device manufacturer, drug manufacturer, device trade name, keyword heading word, floating subheading word, candidate term word] 1815

21 exp Patient Reported Outcome Measures/ 38918

22 patient health questionnaire/ or self report/ 140854

23 12 or 13 or 14 or 15 or 16 or 17 or 18 or 19 or 20 or 21 or 22 869429

24 (clinical adj2 practice).mp. [mp=title, abstract, heading word, drug trade name, original title, device manufacturer, drug manufacturer, device trade name, keyword heading word, floating subheading word, candidate term word] 519762

25 health service*.mp. [mp=title, abstract, heading word, drug trade name, original title, device manufacturer, drug manufacturer, device trade name, keyword heading word, floating subheading word, candidate term word] 616186

26 health care setting.mp. [mp=title, abstract, heading word, drug trade name, original title, device manufacturer, drug manufacturer, device trade name, keyword heading word, floating subheading word, candidate term word] 4013

27 healthcare setting.mp. [mp=title, abstract, heading word, drug trade name, original title, device manufacturer, drug manufacturer, device trade name, keyword heading word, floating subheading word, candidate term word] 4181

28 exp Health Services/ 6142647

29 24 or 25 or 26 or 27 or 28 6342109

30 exp Implementation Science/ 2540

31 implement*.mp. [mp=title, abstract, heading word, drug trade name, original title, device manufacturer, drug manufacturer, device trade name, keyword heading word, floating subheading word, candidate term word] 786211

32 30 or 31 786211

33 11 and 23 and 29 and 32 4160

34 limit 33 to (english language and yr="2009 -Current") 3499
