# Supplementary material for: Implementation of child-centred outcome measures in routine paediatric healthcare practice: a systematic review
Source: Health Qual Life Outcomes. 2023 Jul 3;21:63. doi: 10.1186/s12955-023-02143-9 (PMC10316621; doi:10.1186/s12955-023-02143-9)
Supplement: Supplementary file 3 — Additional file 3. [file 12955_2023_2143_MOESM3_ESM.docx]

## S3: CINHAL Search

CINAHL Friday, March 25, 2022 1:03:29 PM

# Query Limiters/Expanders Last Run Via Results

S34 S10 AND S22 AND S28 AND S31 Limiters - Published Date: 20090101-20221231; English Language

Expanders - Apply equivalent subjects

Search modes - Boolean/Phrase Interface - EBSCOhost Research Databases

Search Screen - Basic Search

Database - CINAHL 1,399

S33 S10 AND S22 AND S28 AND S31 Limiters - Published Date: 20090101-20221231

Expanders - Apply equivalent subjects

Search modes - Boolean/Phrase Interface - EBSCOhost Research Databases

Search Screen - Basic Search

Database - CINAHL 1,406

S32 S10 AND S22 AND S28 AND S31 Expanders - Apply equivalent subjects

Search modes - Boolean/Phrase Interface - EBSCOhost Research Databases

Search Screen - Basic Search

Database - CINAHL 1,767

S31 S29 OR S30 Expanders - Apply equivalent subjects

Search modes - Boolean/Phrase Interface - EBSCOhost Research Databases

Search Screen - Advanced Search

Database - CINAHL 243,622

S30 (MH "Implementation Science") Expanders - Apply equivalent subjects

Search modes - Boolean/Phrase Interface - EBSCOhost Research Databases

Search Screen - Advanced Search

Database - CINAHL 517

S29 implement* Expanders - Apply equivalent subjects

Search modes - Boolean/Phrase Interface - EBSCOhost Research Databases

Search Screen - Advanced Search

Database - CINAHL 243,622

S28 S23 OR S24 OR S25 OR S26 OR S27 Expanders - Apply equivalent subjects

Search modes - Boolean/Phrase Interface - EBSCOhost Research Databases

Search Screen - Advanced Search

Database - CINAHL 1,307,655

S27 (MH "Health Services+") Expanders - Apply equivalent subjects

Search modes - Boolean/Phrase Interface - EBSCOhost Research Databases

Search Screen - Advanced Search

Database - CINAHL 1,081,407

S26 Healthcare setting Expanders - Apply equivalent subjects

Search modes - Boolean/Phrase Interface - EBSCOhost Research Databases

Search Screen - Advanced Search

Database - CINAHL 16,782

S25 Health care setting Expanders - Apply equivalent subjects

Search modes - Boolean/Phrase Interface - EBSCOhost Research Databases

Search Screen - Advanced Search

Database - CINAHL 16,782

S24 Health service* Expanders - Apply equivalent subjects

Search modes - Boolean/Phrase Interface - EBSCOhost Research Databases

Search Screen - Advanced Search

Database - CINAHL 332,555

S23 Clinical N2 practice Expanders - Apply equivalent subjects

Search modes - Boolean/Phrase Interface - EBSCOhost Research Databases

Search Screen - Advanced Search

Database - CINAHL 97,102

S22 S11 OR S12 OR S13 OR S14 OR S15 OR S16 OR S17 OR S18 OR S19 OR S20 OR S21 Expanders - Apply equivalent subjects

Search modes - Boolean/Phrase Interface - EBSCOhost Research Databases

Search Screen - Advanced Search

Database - CINAHL 268,394

S21 (MH "Self Report+") Expanders - Apply equivalent subjects

Search modes - Boolean/Phrase Interface - EBSCOhost Research Databases

Search Screen - Advanced Search

Database - CINAHL 79,678

S20 (MH "Patient-Reported Outcomes+") Expanders - Apply equivalent subjects

Search modes - Boolean/Phrase Interface - EBSCOhost Research Databases

Search Screen - Advanced Search

Database - CINAHL 4,176

S19 Proxy N2 measure Expanders - Apply equivalent subjects

Search modes - Boolean/Phrase Interface - EBSCOhost Research Databases

Search Screen - Advanced Search

Database - CINAHL 1,044

S18 Self N2 measure Expanders - Apply equivalent subjects

Search modes - Boolean/Phrase Interface - EBSCOhost Research Databases

Search Screen - Advanced Search

Database - CINAHL 14,547

S17 Patient cent#red outcome measure* Expanders - Apply equivalent subjects

Search modes - Boolean/Phrase Interface - EBSCOhost Research Databases

Search Screen - Advanced Search

Database - CINAHL 258

S16 Patient reported outcome* Expanders - Apply equivalent subjects

Search modes - Boolean/Phrase Interface - EBSCOhost Research Databases

Search Screen - Advanced Search

Database - CINAHL 18,118

S15 PCOM Expanders - Apply equivalent subjects

Search modes - Boolean/Phrase Interface - EBSCOhost Research Databases

Search Screen - Advanced Search

Database - CINAHL 122

S14 PROM Expanders - Apply equivalent subjects

Search modes - Boolean/Phrase Interface - EBSCOhost Research Databases

Search Screen - Advanced Search

Database - CINAHL 2,520

S13 PRO Expanders - Apply equivalent subjects

Search modes - Boolean/Phrase Interface - EBSCOhost Research Databases

Search Screen - Advanced Search

Database - CINAHL 31,648

S12 Symptom measure* Expanders - Apply equivalent subjects

Search modes - Boolean/Phrase Interface - EBSCOhost Research Databases

Search Screen - Advanced Search

Database - CINAHL 14,219

S11 Outcome measure* Expanders - Apply equivalent subjects

Search modes - Boolean/Phrase Interface - EBSCOhost Research Databases

Search Screen - Advanced Search

Database - CINAHL 140,802

S10 S1 OR S2 OR S3 OR S4 OR S5 OR S6 OR S7 OR S8 OR S9 Expanders - Apply equivalent subjects

Search modes - Boolean/Phrase Interface - EBSCOhost Research Databases

Search Screen - Advanced Search

Database - CINAHL 1,351,701

S9 (MH "Infant+") Expanders - Apply equivalent subjects

Search modes - Boolean/Phrase Interface - EBSCOhost Research Databases

Search Screen - Advanced Search

Database - CINAHL 276,577

S8 (MH "Adolescence+") Expanders - Apply equivalent subjects

Search modes - Boolean/Phrase Interface - EBSCOhost Research Databases

Search Screen - Advanced Search

Database - CINAHL 573,077

S7 (MH "Child+") Expanders - Apply equivalent subjects

Search modes - Boolean/Phrase Interface - EBSCOhost Research Databases

Search Screen - Advanced Search

Database - CINAHL 719,323

S6 Infant Expanders - Apply equivalent subjects

Search modes - Boolean/Phrase Interface - EBSCOhost Research Databases

Search Screen - Advanced Search

Database - CINAHL 319,180

S5 Young people Expanders - Apply equivalent subjects

Search modes - Boolean/Phrase Interface - EBSCOhost Research Databases

Search Screen - Advanced Search

Database - CINAHL 24,330

S4 Teen* Expanders - Apply equivalent subjects

Search modes - Boolean/Phrase Interface - EBSCOhost Research Databases

Search Screen - Advanced Search

Database - CINAHL 23,972

S3 Adolescen* Expanders - Apply equivalent subjects

Search modes - Boolean/Phrase Interface - EBSCOhost Research Databases

Search Screen - Advanced Search

Database - CINAHL 610,093

S2 P#ediatric* Expanders - Apply equivalent subjects

Search modes - Boolean/Phrase Interface - EBSCOhost Research Databases

Search Screen - Advanced Search

Database - CINAHL 210,717

S1 Child* Expanders - Apply equivalent subjects

Search modes - Boolean/Phrase Interface - EBSCOhost Research Databases

Search Screen - Advanced Search

Database - CINAHL 876,839
