# Supplementary material for: Implementation of child-centred outcome measures in routine paediatric healthcare practice: a systematic review
Source: Health Qual Life Outcomes. 2023 Jul 3;21:63. doi: 10.1186/s12955-023-02143-9 (PMC10316621; doi:10.1186/s12955-023-02143-9)
Supplement: Supplementary file 4 — Additional file 4. [file 12955_2023_2143_MOESM4_ESM.docx]

## S4: PsycInfo Search

APA PsycInfo <1806 to March Week 3 2022>

1 Child*.mp. [mp=title, abstract, heading word, table of contents, key concepts, original title, tests & measures, mesh word] 860850

2 P?ediatric*.mp. [mp=title, abstract, heading word, table of contents, key concepts, original title, tests & measures, mesh word] 55446

3 adolescen*.mp. [mp=title, abstract, heading word, table of contents, key concepts, original title, tests & measures, mesh word] 498236

4 teen*.mp. [mp=title, abstract, heading word, table of contents, key concepts, original title, tests & measures, mesh word] 25180

5 young people.mp. [mp=title, abstract, heading word, table of contents, key concepts, original title, tests & measures, mesh word] 32549

6 infant.mp. [mp=title, abstract, heading word, table of contents, key concepts, original title, tests & measures, mesh word] 93024

7 exp Pediatrics/ 33034

8 1 or 2 or 3 or 4 or 5 or 6 or 7 1186159

9 outcome measure*.mp. [mp=title, abstract, heading word, table of contents, key concepts, original title, tests & measures, mesh word] 42817

10 symptom measure*.mp. [mp=title, abstract, heading word, table of contents, key concepts, original title, tests & measures, mesh word] 1114

11 PRO.mp. [mp=title, abstract, heading word, table of contents, key concepts, original title, tests & measures, mesh word] 20124

12 PCOM.mp. [mp=title, abstract, heading word, table of contents, key concepts, original title, tests & measures, mesh word] 15

13 PROM.mp. [mp=title, abstract, heading word, table of contents, key concepts, original title, tests & measures, mesh word] 381

14 patient reported outcome*.mp. [mp=title, abstract, heading word, table of contents, key concepts, original title, tests & measures, mesh word] 4097

15 patient cent?red outcome measure*.mp. [mp=title, abstract, heading word, table of contents, key concepts, original title, tests & measures, mesh word] 44

16 (self adj2 measure).mp. [mp=title, abstract, heading word, table of contents, key concepts, original title, tests & measures, mesh word] 11056

17 (proxy adj2 measure).mp. [mp=title, abstract, heading word, table of contents, key concepts, original title, tests & measures, mesh word] 595

18 exp Patient Reported Outcome Measures/ 498

19 patient health questionnaire/ or self report/ 20774

20 exp Measurement/ 488424

21 9 or 10 or 11 or 12 or 13 or 14 or 15 or 16 or 17 or 18 or 19 or 20 562630

22 (clinical adj2 practice).mp. [mp=title, abstract, heading word, table of contents, key concepts, original title, tests & measures, mesh word] 65115

23 health service*.mp. [mp=title, abstract, heading word, table of contents, key concepts, original title, tests & measures, mesh word] 135023

24 health care setting.mp. [mp=title, abstract, heading word, table of contents, key concepts, original title, tests & measures, mesh word] 1045

25 healthcare setting.mp. [mp=title, abstract, heading word, table of contents, key concepts, original title, tests & measures, mesh word] 675

26 exp Clinical Practice/ 22587

27 exp Health Care Services/ 228798

28 22 or 23 or 24 or 25 or 26 or 27 324667

29 implement*.mp. [mp=title, abstract, heading word, table of contents, key concepts, original title, tests & measures, mesh word] 202756

30 8 and 20 and 28 and 29 1005

31 limit 30 to (english language and yr="2009 -Current") 790
